# Characterizing the Self-Assembly Properties of Monoolein Lipid Isosteres

**DOI:** 10.1021/acs.jpcb.2c07215

**Published:** 2023-02-16

**Authors:** Alessandro Fracassi, Kira A. Podolsky, Sudip Pandey, Cong Xu, Joshua Hutchings, Soenke Seifert, Carlos R. Baiz, Sunil K. Sinha, Neal K. Devaraj

**Affiliations:** † Department of Chemistry and Biochemistry, University of California, San Diego, 9500 Gilman Drive, Natural Sciences Building 3328, La Jolla, California92093, United States; ‡ Department of Physics, University of California, San Diego, 9500 Gilman Drive, Mayer Hall Addition 4561, La Jolla, California92093, United States; § Department of Chemistry, The University of Texas at Austin, 105 E. 24th St. Stop A5300, Austin, Texas78712−1224, United States; ∥ Department of Molecular Biology, School of Biological Sciences, University of California, San Diego, La Jolla, California92093, United States; ⊥ X-ray Science Division, Argonne National Laboratory, 9700 South Cass Avenue, Argonne, Illinois60439, United States

## Abstract

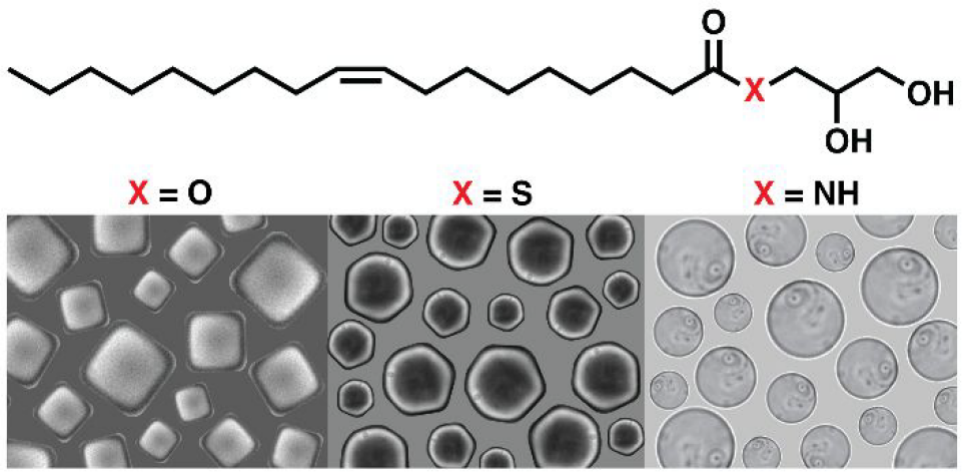

Living cells feature lipid compartments which exhibit a variety of shapes and structures that assist essential cellular processes. Many natural cell compartments frequently adopt convoluted nonlamellar lipid architectures that facilitate specific biological reactions. Improved methods for controlling the structural organization of artificial model membranes would facilitate investigations into how membrane morphology affects biological functions. Monoolein (MO) is a single-chain amphiphile which forms nonlamellar lipid phases in aqueous solution and has wide applications in nanomaterial development, the food industry, drug delivery, and protein crystallization. However, even if MO has been extensively studied, simple isosteres of MO, while readily accessible, have seen limited characterization. An improved understanding of how relatively minor changes in lipid chemical structure affect self-assembly and membrane topology could instruct the construction of artificial cells and organelles for modeling biological structures and facilitate nanomaterial-based applications. Here, we investigate the differences in self-assembly and large-scale organization between MO and two MO lipid isosteres. We show that replacing the ester linkage between the hydrophilic headgroup and hydrophobic hydrocarbon chain with a thioesther or amide functional group results in the assembly of lipid structures with different phases not resembling those formed by MO. Using light and cryo-electron microscopy, small-angle X-ray scattering, and infrared spectroscopy, we demonstrate differences in the molecular ordering and large-scale architectures of the self-assembled structures made from MO and its isosteric analogues. These results improve our understanding of the molecular underpinnings of lipid mesophase assembly and may facilitate the development of MO-based materials for biomedicine and as model lipid compartments.

## Introduction

Lipid membranes of living cells can assemble into various shapes and morphologies to govern and regulate biological functions. While cell membranes are largely composed of lamellar structures, nonlamellar phases have also been observed^1,2^ and can promote unique local architectures that facilitate cellular functions such as fission and fusion.^3,4^ Some subcellular compartments can exhibit structural rearrangements from lamellar to nonlamellar phases in response to specific conditions. For instance, etioplasts in germinating plant cells adopt a nonlamellar membrane structure in the dark which transforms into the lamellar thylakoid membrane, or photosynthetic membrane, in response to light irradiation.^5,6^ The membrane of the smooth endoplasmic reticulum has been shown to form cubic phases during membrane protein expression, viral infection, and drug detoxification.^7,8^ Given the relevance of nonlamellar lipid compartments in cells, developing nonlamellar biomaterials as artificial cell models would be useful for understanding the molecular underpinnings of cellular functions.

Nonlamellar lipid phases are useful biomaterials as they self-assemble in water, generating structures with accessible hydrophilic and hydrophobic environments. As such, nonlamellar lipid phases have found widespread application in structural biology and biomedicine.^9,10^ The most common nonlamellar phases are the lipidic cubic, hexagonal, and sponge phases.^11^ Nonlamellar lipid mesophases have been exploited as drug delivery systems and food emulsifiers due to their biocompatibility, nontoxicity, and stability in excess water.^12−18^ They have also been used frequently in structural biology to promote the crystallization of membrane proteins.^19,20^ Additionally, the ability of some mesophases to encapsulate and recruit hydrophilic or hydrophobic molecules while maintaining their structural integrity has led to their use as nanoreactors for chemical reactions and enzyme immobilization.^21−28^

One of the most widely used lipids for the formation of nonlamellar lipidic cubic phases is 1-(*cis*-9-octadecenoyl)-*rac*-glycerol, or 1-monoolein (MO).^29^ As a monoacylglycerol, MO is an uncharged single-chain amphiphile composed of a hydrophobic tail and hydrophilic headgroup connected by an ester linkage. MO can self-assemble into liquid crystalline mesophases when dispersed in aqueous media. However, due to the absence of charge and steric repulsion, the dispersed particles phase separate rapidly to form a bulk cubic phase in excess water. To obtain a stable particle dispersion, nonionic block copolymers such as Pluronic 127 are commonly used, preventing aggregation and imparting stabilization by steric means.^30,31^

Most previous studies have focused on creating MO nonlamellar nanostructures.^32,33^ Physical means, such as ultrasonication, are used to generate nanosized aggregates. In contrast, the formation of micrometer-sized nonlamellar assemblies would mimic subcellular mesophase structures and could serve as artificial organelles.^34−36^ In either case, it would be beneficial to understand how small changes in the chemical structure, independent of environmental conditions, can determine large-scale organizational changes in MO-based materials. Indeed, it has been demonstrated that different 3D molecular architectures confer unique macroscopic properties and functionalities on lipid assemblies.^2^ For this reason, many studies have examined how the macrostructures of lipid assemblies depend on length,^37,38^ chain splay,^39^ and branching of their hydrophobic tails^40^ or on the size and charge of the polar headgroups.^41,42^ However, only a few studies have investigated the effect of changing the nature of the chemical linkage between the polar head and the alkyl tail. In particular, replacing a single atom, such as an oxygen in the ester linkage, may dramatically influence the hydrogen bonding and size of the headgroup. This could result in alternative biophysical interactions at the water–lipid interface and in between lipid molecules, leading to structural changes in the lipid assemblies.

In this work, we demonstrate that changing the MO linkage from an ester to a thioester or amide significantly affects the large-scale structures that spontaneously assemble in water. We characterized the lipid assemblies using light microscopy, cryo-electron microscopy (cryo-EM), small-angle X-ray scattering (SAXS), and Fourier transform infrared spectroscopy (FTIR) to evaluate the molecular ordering of the lipidic phase structures. Furthermore, we assessed the stability of each MO derivative at varying temperatures. Analysis of the morphological differences of the structures formed from MO analogues provides insight into how simple isosteric modifications in small molecule structure can affect the organization and stability of the formed aggregates.

## Methods

### Chemicals and Materials

All reagents and solvents were purchased from Sigma-Aldrich Co. LLC (Merck KGaA, Darmstadt, Germany) unless indicated otherwise. Water was purified by a Millipore purification system (Merck KGaA). NMR spectra were recorded on a Jeol ECA-500 (Jeol Ltd., Tokyo, Japan). Solvent mixtures for chromatography are reported as volume/volume (v/v) ratios. Reverse phase high performance liquid chromatography (HPLC) purifications were performed on a CombiFlash EZ Prep (Teledyne Isco, Inc.) with a RediSep Prep C18 column (20 × 150 mm^2^) with Phase A/Phase B gradients [Phase A: H_2_O with 0.1% formic acid; Phase B: MeOH with 0.1% formic acid] at a flow rate of 19 mL/min. The HPLC was equipped with a UV–vis detector and an evaporative light scattering detector (ELSD). High-resolution mass (HRMS) analyses were performed by electrospray ionization–time of flight (ESI-TOF) on an Agilent 6230 Accurate-Mass TOF-MS mass spectrometer.

### Synthesis of Compound **2**

Compound **2** was synthesized according to Scheme S1. To a solution of oleic acid (**S1**, 100 mg, 0.35 mmol, 1.0 equiv) in 2 mL of DMF, DIPEA (457.6 mg, 3.5 mmol, 10.0 equiv) and HATU (133.6 mg, 0.35 mmol, 1.0 equiv) were added, and the solution was stirred at rt for 5 min. Subsequently, compound **S2** (189.3 mg, 1.75 mmol, 5.0 equiv) was added to the mixture, and the reaction was stirred for 1 h at rt under Ar. After removal of DMF under reduced pressure, the crude mixture was purified by reverse phase HPLC (solvent: ACN–H_2_O (50:50 for 2 min, to 90:10 in 20 min, and kept at 90:10 for 10 min) in the presence of 0.1% trifluoroacetic acid), providing the pure compound **2** as a white solid (46.9 mg, 0.126 mmol, yield = 36%). ^1^H NMR (500 MHz, in CDCl_3_): δ 5.38–5.26 (m, 2H), 3.81 (quin, *J* = 5.2 Hz, 1H), 3.70–3.48 (m, 2H), 3.14–2.97 (m, 2H), 2.60 (t, *J* = 7.4 Hz, 2H), 2.04–1.94 (m, 4H), 1.66 (quin, *J* = 7.2 Hz, 2H), 1.35–1.22 (m, 20H), 0.88 (t, *J* = 6.5 Hz, 3H). ^13^C NMR (125 MHz, in CDCl_3_): δ 201.7, 130.2, 129.8, 77.3, 71.3, 64.5, 44.2, 32.0, 31.6, 29.9, 29.8, 29.6, 29.4, 29.2, 29.1, 29.0, 27.3, 27.2, 25.8, 22.8, 14.2. MS (ESI^+^) *m*/*z*: [M + H]^+^ calculated for C_21_H_41_O_3_S^+^, 373.2771; found, 373.2771.

### Synthesis of Compound **3**

Compound **3** was synthesized according to Scheme S2. To a solution of oleic acid (**S1**, 310.7 mg, 1.10 mmol, 1.0 equiv) in 2 mL of DMF, DIPEA (284.4 mg, 2.20 mmol, 2.0 equiv) and HATU (380.2 mg, 1.10 mmol, 1.0 equiv) were added, and the solution was stirred at rt for 5 min. Subsequently, compound **S2** (100.0 mg, 1.10 mmol, 1.0 equiv) was added to the mixture, and the reaction was stirred for 1 h at rt under Ar. After removal of DMF under reduced pressure, the crude mixture was purified by reverse phase HPLC (solvent: MeOH–H_2_O (50:50 for 2 min, to 95:5 in 3 min, and kept at 95:5 for 10 min) in the presence of 0.1% formic acid), providing the pure compound **3** as a white wax (282.0 mg, 0.79 mmol, yield = 72%). ^1^H NMR (500 MHz, in CDCl_3_): δ 6.32 (t, *J* = 6.2 Hz, 1H), 5.38–5.29 (m, 2H), 3.77 (quin, *J* = 5.1 Hz, 1H), 3.61–3.50 (m, 2H), 3.49–3.29 (m, 4H), 2.22 (t, *J* = 7.5 Hz, 2H), 2.04–1.95 (m, 4H), 1.62 (quin, *J* = 7.2 Hz, 2H), 1.37–1.20 (m, 20H), 0.87 (t, *J* = 6.6 Hz, 3H). ^13^C NMR (125 MHz, in CDCl_3_): δ 175.7, 130.2, 129.8, 71.2, 63.6, 42.3, 36.7, 32.0, 29.9, 29.8, 29.7, 29.5, 29.4, 29.3, 27.4, 27.3, 25.6, 22.8, 14.3. MS (ESI^+^) *m*/*z*: [M + H]^+^ calculated for C_21_H_42_NO_3_^+^, 356.3159; found, 356.3158.

### General Preparation Protocol of Lipid Dispersions

The lipids were weighed and dissolved in MeOH in a glass vial to obtain a 50 mM stock solution. The desired amount of lipid in MeOH was transferred to another glass vial, and a thin lipid film was created by carefully evaporating the solvent under Ar. The required volume of a solution of Pluronic F127 in water (15 μM) was added to achieve a final lipid concentration of 10 mM. The sample was vortexed vigorously, heated at 75 °C for 2 min, and vortexed again, until all lipids were completely dispersed to form a turbid white suspension.

### Cryo-Electron Microscopy (Cryo-EM)

EM grids (Lacey Carbon Film, Electron Microscopy Sciences #LC200-Cu) were glow-discharged (Emitech K350 unit at 20 mA for 30 s), deposited with 4 μL of lipid dispersion, blotted, and then plunged into liquid ethane using a Vitrobot (Mark IV, Thermo Fisher Scientific). Images were collected on a Titan Krios G3 (Thermo Fisher Scientific) operated at 300 keV equipped with a K3 detector and a 1067HD BioContinuum energy filter (Gatan) with a 15 eV slit width. Dose-fractionated images were acquired using SerialEM^43^ in low dose counting mode with a total dose of 50 e/Å^2^ at 2.16 Å/pixel and 4 μm nominal defocus. Images were motion- and CTF-corrected in Warp.^44^ Representative images were prepared in ImageJ.^45^

### Small-Angle X-ray Scattering (SAXS)

SAXS measurements were performed at the 12-ID-B beamline from the Argonne Photon Source synchrotron. The X-ray energy was 18 keV. The wavevector transfer is defined as *q* = 4π sin(θ)/λ, where 2θ is the angle between the incident and the scattered X-rays and λ is the wavelength. The distance between the sample and the detector was 2269.44 mm. The samples were filled in glass capillaries with a diameter of 1 mm and a wall thickness of 0.01 mm purchased from Hampton Research. We measured each sample for 1 s at 5 different positions. The experiments were performed at 10, 25, 37, and 50 °C. We reduced the 2D data to a 1D scattering for easier analysis by circular averaging. A background sample composed of H_2_O was subtracted from all the samples. All data were fitted with Gaussian peaks on top of a flat background. All data analysis was performed using the Python programming software.

### Fluorescence Spectroscopy of Laurdan

The fluorescence spectra of dispersions of compound **1**, **2**, or **3**, containing 0.05 mol % of 6-lauroyl-2-(dimethylamino)naphthalene (Laurdan) were recorded at four different temperatures (10, 25, 37, and 50 °C) using a Cary Eclipse fluorescence spectrofluorometer (Agilent) (λ_ex_ = 360 nm; λ_em_ = 400–600 nm). Samples were prepared following the same protocol described above, but adding 0.05 mol % Laurdan from a 10 mM stock solution in MeOH for the preparation of the lipid film.

### Turbidimetry Measurements

The stability of the lipid dispersions was assessed by following the time-dependent changes of the optical density at 600 nm (OD_600_), with a Jasco J-1500 circular dichroism spectrometer, using quartz cuvettes of 0.1 cm path length. Lipid dispersions were prepared as described above and immediately incubated at the desired temperature. The OD_600_ was measured every 15 min over 3 h and at 12, 24, and 48 h at four different temperatures (10, 25, 37, and 50 °C).

### Visual Assessment of Lipid Dispersion Stability

Visual inspection of lipid dispersions was performed through observation of the samples after 3, 12, 24, and 48 h at four different temperatures (10, 25, 37, and 50 °C). All samples were prepared according to the protocol described above and immediately incubated at the desired temperature.

### Fourier Transform IR (FTIR) Spectroscopy

FTIR spectra of MO **1** and its thioester **2** and amide **3** analogues in D_2_O were measured at 0.5 cm^–1^ resolution using a Bruker Invenio spectrometer. The sample enclosure was purged with dry air to remove absorption peaks from water vapor. Each spectrum is an average of 32 scans from 1000 to 4000 cm^–1^. The sample was equilibrated for 10 min at each temperature set point using a custom-built sample cell connected to a recirculating water chiller, which provides a temperature accuracy of 0.1 °C. Spectra were measured at the following temperatures: 10, 15, 20, 25, 30, 35, 40, 45, and 50 °C. Experiments were repeated at least three times to ensure reproducibility.

Time series FTIR spectra were measured using freshly dissolved amide **3**, which was heated at 50 °C for 20 min. The sample cell was then incubated at 25 °C, and FTIR spectra were collected every 5 min. Because the heat that the sample stores is negligible compared to the recirculating system and the thermal conduction between the two is efficient, the temperature of the sample reached 25 °C in <3 min. Each spectrum was captured at 25 °C in the experiment.

## Results and Discussion

**Figure 1:**
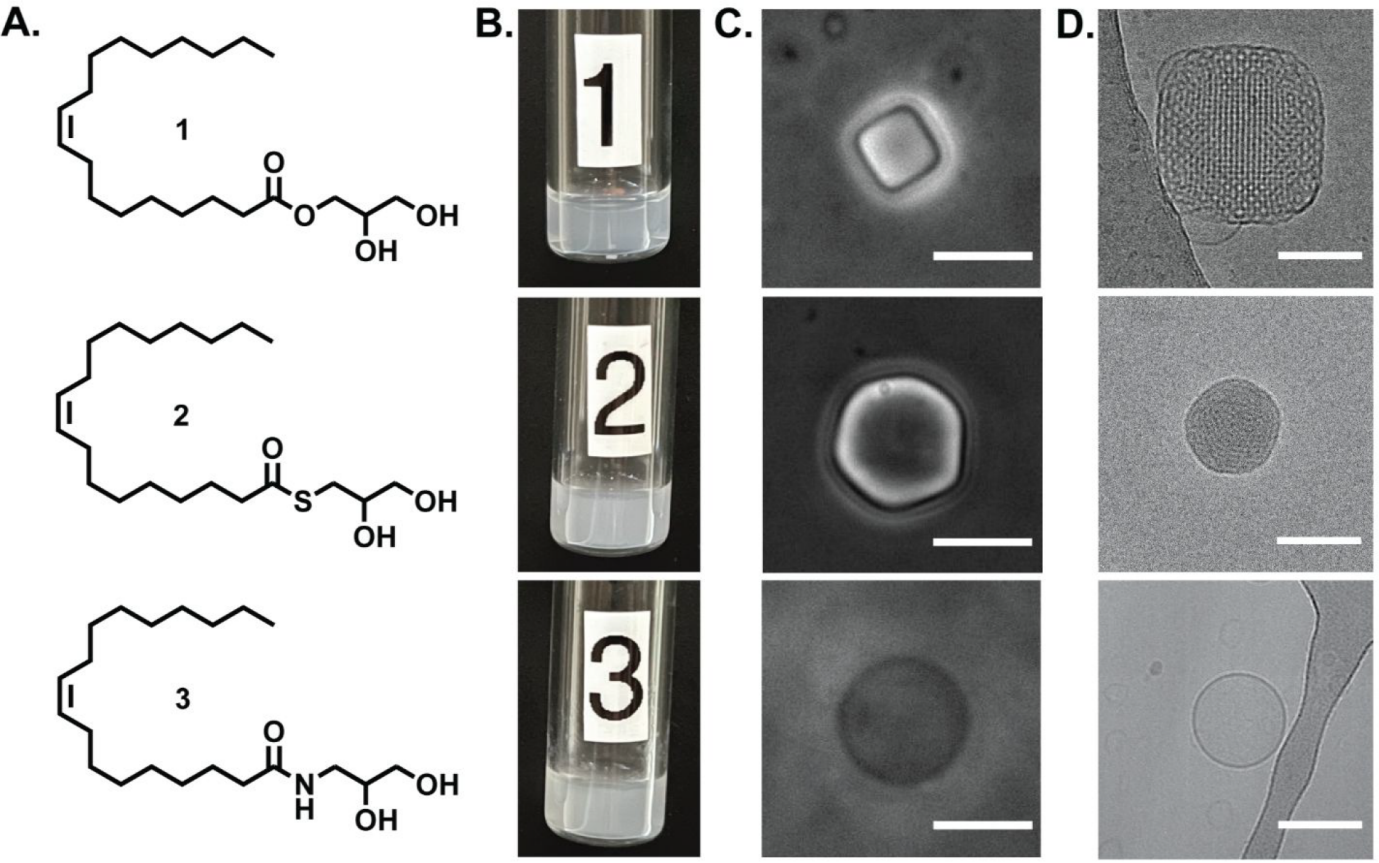
Structural characterization of the lipid dispersions. (A) Chemical structures of MO **1**, its thioester **2**, and amide **3** analogues. (B) Lipid dispersion of MO **1**, thioester **2**, and amide **3**. (C) Phase contrast microscopy images of the lipid dispersions (scale bar 10 μm). (D) Cryo-EM images of the lipid dispersions (scale bar 100 nm). Positions throughout the image are as follows: MO **1** (top), thioester analogue **2** (middle), and amide analogue **3** (bottom).

We initially tested the effect of modifying the linker functionality between the glycerol headgroup and oleyl tail of MO (compound **1**) by substituting the ester with a thioester (compound **2**)^46,47^ or amide (compound **3**). We synthesized the appropriate lipid analogue of MO **1** and upon lipid film hydration obtained a dispersion of lipid in aqueous media containing Pluronic F127 as a stabilizer (Figure 1A,B). To create uniformly turbid dispersions, the samples were vigorously vortexed, heated at 75 °C, and vortexed again (Figure 1B). To obtain micrometer-sized structures, we avoided the ultrasonication step usually performed during the preparation of nanometer-sized mesophases of MO dispersions.^48^

Phase contrast microscopy analysis of the dispersions at room temperature showed dramatic differences in the microscale structure formed from MO and its analogues. In agreement with previous works,^49,50,9^ dispersions of MO **1** at 25 °C mostly consisted of micrometer-sized structures with distinct cubic shapes and defined edges (Figures 1C, top). Observing the structures from compound **2** showed the coexistence of well-defined hexagonal structures (Figure 1C, middle) and assemblies with less defined edges (Figure S5). Therefore, the ability of compound **2** to form hexagonal phase particles was unclear from light microscopy alone. Dispersions of compound **3** contained an abundance of multilamellar vesicles (Figure 1C, bottom) as well as rounded structures resembling other nonlamellar phases, such as lipid sponge phase droplets (Figure S6).

To better visualize the morphologies and internal structures of the particles formed, we employed cryo-EM (Figure 1D), which allows to visualize the smallest subset of particles in the dispersions. Cryo-EM confirmed the presence of squared particles with a well-defined internal cubic lattice in MO **1** dispersions (Figure 1D, top). Interestingly, cryo-EM analysis of compound **2** showed the abundance of particles with imperfectly defined hexagonal edges (Figure 1D, middle) or completely round shapes (Figure S7), similar to what was observed by phase contrast microscopy. Additionally, the presence of an internal structural periodicity was clear and strongly suggests an inverse hexagonal phase arrangement within both hexagonal and rounded particle populations. Indeed, lipid particles with hexagonal phase can display a rounded shaped architecture characterized by the presence of curved striations that represent deformed water cylinders.^51,10^ Finally, cryo-EM analysis of compound **3** confirmed the presence of lamellar vesicles (Figure 1D, bottom).

**Figure 2:**
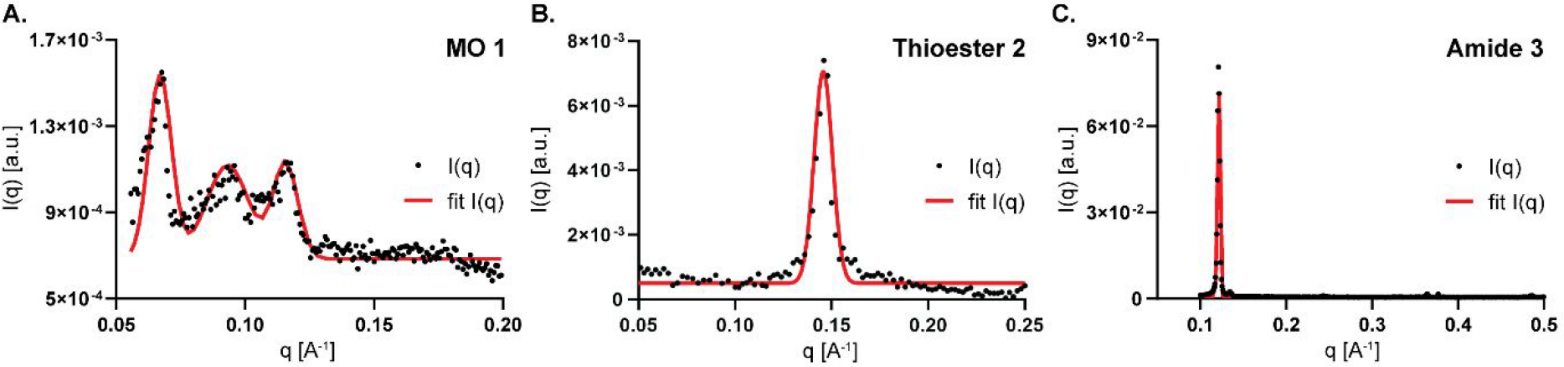
Synchrotron SAXS intensity profiles (black circles) recorded at 25 °C with fitting (solid red line) of lipid dispersions of (A) MO **1**, (B) thioester **2**, and (C) amide **3**.

To determine the phase type and structural parameters of the particles formed in the lipid dispersions, we performed SAXS measurements at the Advanced Photon Source (APS) synchrotron. Figure 2 displays the SAXS pattern of lipid dispersion of compounds **1**, **2**, and **3** at 25 °C, indicating that the three formulations contain three different lipid architectures. Similarly to previous reports, the SAXS profile of dispersions of compound **1** showed three distinct Bragg peaks.^52,53^ From the ratios of the fitted *q* values of the positions of the second and third peaks to that of the first peak (√2 and √3, respectively), we deduced that the structure corresponded to the *Im*3*m* cubic space group. The SAXS profile of compound **2** shows a single intense peak alongside a broad distribution, which makes the phase identification difficult from SAXS alone. The SAXS profile of dispersions of compound **3** showed sharp Bragg reflections characteristic of the long-range positional order of lamellar phases, L_α_. However, the higher order peaks had very small intensity compared to the first peak, indicating an unusual bilayer electron density profile (Figure S9).

**Figure 3:**
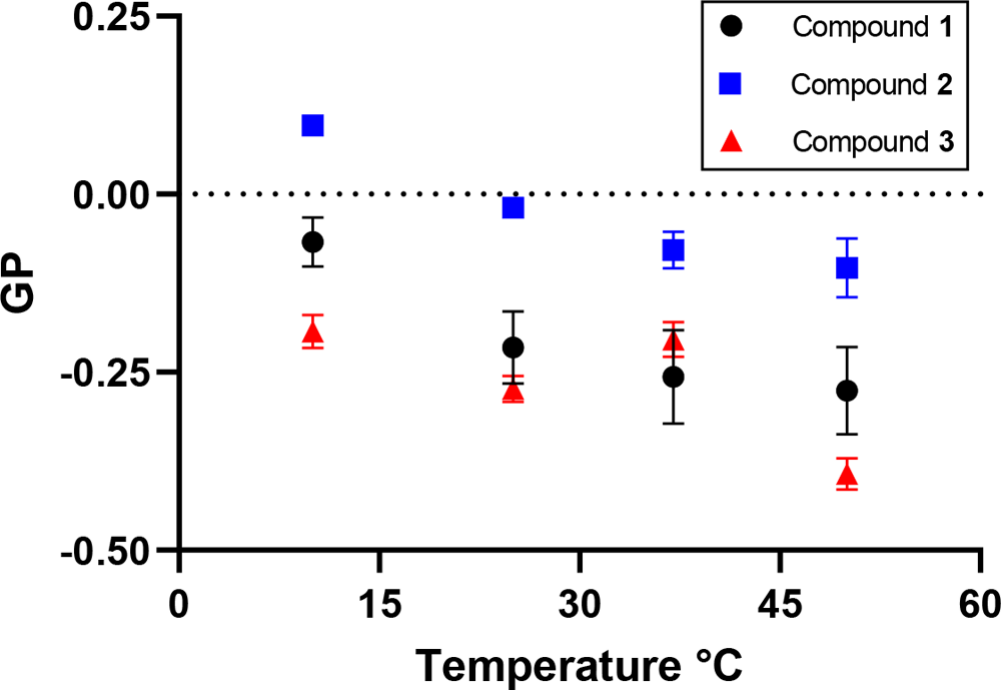
Laurdan GP values at different temperatures (10, 25, 37, and 50 °C) for dispersions of MO **1** (black circles), thioester **2** (blue squares), and amide **3** (red triangles) containing 0.05 mol % Laurdan.

To further improve our understanding of the molecular organization of the lipid assemblies, we monitored the fluorescence emission of the solvatochromic dye 6-dodecanoyl-*N*,*N*-dimethyl-2-naphthylamine (Laurdan) when added to the lipid dispersions. Laurdan is a fluorescent dye typically used to probe changes in lipid membrane phase.^54−56^ The excited state of Laurdan possesses a dipole moment that renders it very sensitive to the presence of polar compounds in the lipid environment, such as water molecules. Changes from hydrophobic to hydrophilic environments, or low to high polarity, cause a significant red-shift of the fluorescence emission peak observed.^54^ The polarity changes in a lipid phase can be assessed by determining Laurdan generalized polarization (GP), which is defined as
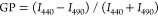
where *I*_440_ and *I*_490_ are the fluorescence intensities at 440 and 490 nm, respectively. Low polarity is associated with high GP values, while high polarity is associated with low GP values. To evaluate the polarity of the lipid phases formed, we determined the GP of Laurdan added to the lipid dispersions at different temperatures. Increasing the temperature can generally result in an increase of the membrane permeability to water, resulting in an increase in the polar environment around the Laurdan probe.^54^ This behavior is clearly visible both for MO **1** and compound **2** (Figure 3). Furthermore, the negative GP values obtained suggest that a general hydrophilic environment is present in the structures formed from compounds **1** and **3** across all the observed temperatures. Conversely, compound **2** showed higher values of GP, indicating a more hydrophobic environment than compounds **1** and **3** overall. Interestingly, in compound **2**, a clear red-shift is observed from 10 to 25 °C, and the fluorescence maximum shifts from 440 to 490 nm at 37 °C (Figure S11), indicating a transition from a largely hydrophobic environment to a hydrophilic one at this temperature (GP transitions from positive to negative, as shown in Figure 3). Compound **3** presents a more complex situation. While its GP does not appreciably change from 10 to 37 °C and instead exhibits irregular shifts, a significant decrease is observed when going from 37 to 50 °C. Taken together, this suggests that insoluble aggregates might be forming at lower temperatures, while a more stable dispersion can form at 50 °C. Thus, replacing the ester functional group affects not only the morphology of the assemblies formed but also their stability.

We next decided to investigate the stability of the lipid dispersions over time at four different temperatures (10, 25, 37, and 50 °C). The stability was evaluated by visual inspection and turbidimetry measurements, which monitor the dispersions optical density at 600 nm (OD_600_) for 48 h. All samples were identically prepared and initially formed suspensions with a milky white consistency with no visible aggregates. The samples were then immediately incubated at the desired temperature. Stability data showed that while MO **1** formed stable dispersions at all the conditions tested, the stability of dispersions of compounds **2** and **3** varied with temperature. For instance, an insoluble precipitate was clearly visible at the bottom of the analysis vessel after 3 h at 10 °C for compounds **2** and **3**. The amount of precipitate steadily increased over time (Figure S25). However, at higher temperatures compounds **2** and **3** showed remarkably diverging behaviors. The stability of compound **2** dispersions notably increased, and the formation of a white precipitate could be observed only after 48 h at 25 °C (Figure S26), while no precipitate was observed at 37 and 50 °C (Figures S27 and S28). Conversely, compound **3** dispersions remained unstable at 25 and 37 °C, resulting in the formation of a white precipitate or crystals within 3 h (Figures S26 and S27). Interestingly, at 50 °C compound **3** formed visually stable dispersions with no formation of any visible precipitate over 48 h (Figure S28).

**Figure 4:**
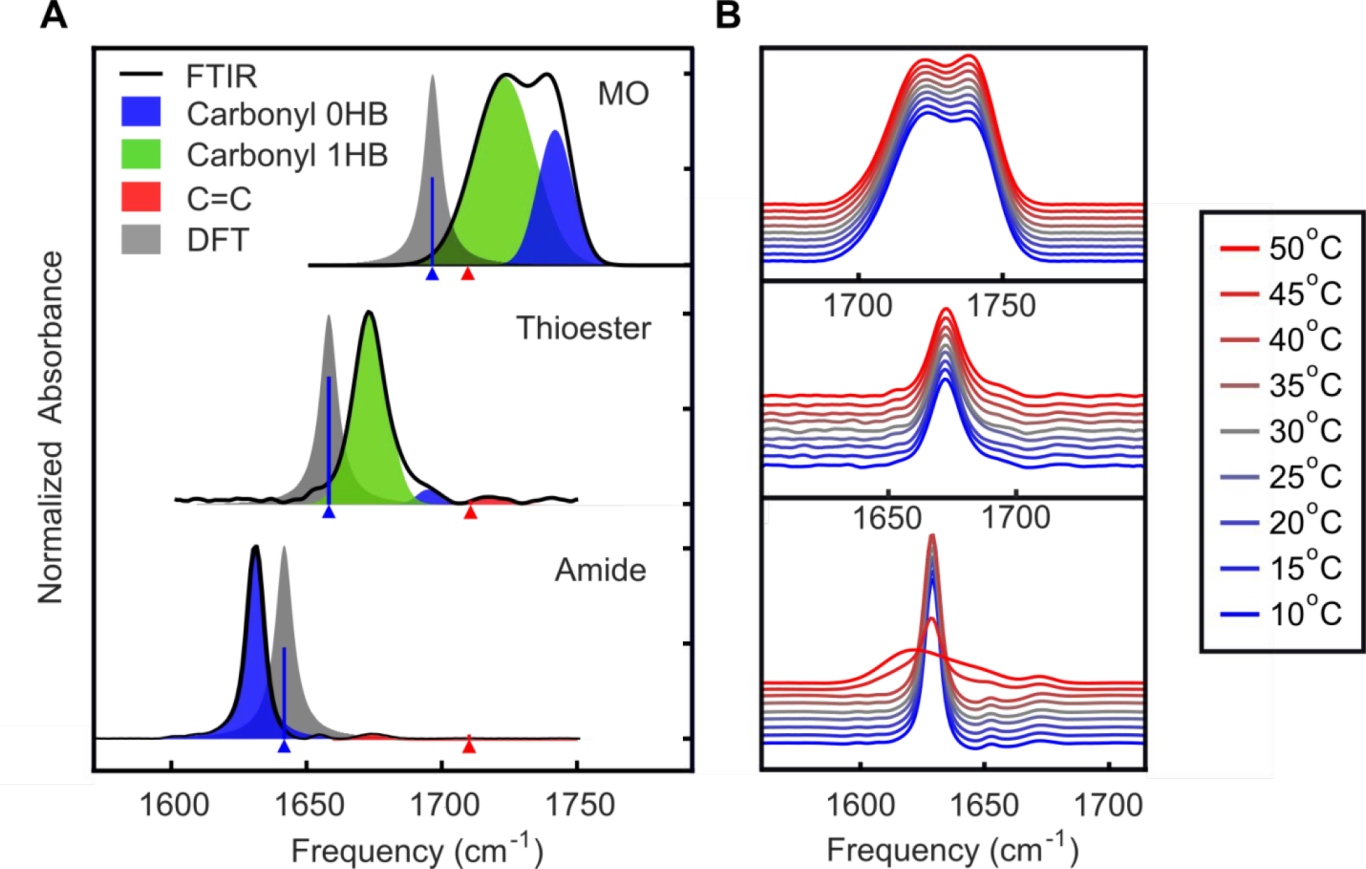
Spectroscopic characterization of MO **1**, thioester **2**, and amide **3**. (A) Experimental FTIR spectrum and peak assignment of MO **1**, thioester **2**, and amide **3** carbonyl stretch at 25 °C based on electronic structure calculations. The C=C bond position partially overlaps with the carbonyl 0HB peak in MO **1**, but it is distinguishable for compounds **2** and **3**. (B) Normalized FTIR spectra of carbonyl from 5 to 50 °C. MO **1** and thioester **2** remain stable across the temperature range measured, whereas the amide **3** analogue exhibits marked changes in line shape, implying possible formation of insoluble aggregates below 45 °C.

To better understand the differences at the lipid–solvent interface, or interfacial environments, of each lipid dispersion, we characterized the C=O stretch mode of the three lipids using FTIR spectroscopy. In general, an oscillator in a water-exposed heterogeneous environment will exhibit a broader peak than one in a homogeneous environment as a result of the larger range of configurational states in the disordered environment. Figure 4A shows experimental spectra (black), fits to the line shapes (shaded areas) and computed peak frequencies (triangles). The differences in line shape suggest that MO analogues exhibit significantly different interfacial environments at 25 °C.^57^ In MO **1**, it can be well represented by a combination of two Gaussian peaks, assigned to 0-Hbond (0HB, blue) and 1-Hbond (1HB, green) ensembles. In the thioester **2**, carbonyl 0-Hbond (blue), the C=C double bond stretch (red) and carbonyl 1-Hbond (green) are represented by Gaussian peaks, suggesting inhomogeneous broadening.^58,59^ For compound **3**, one single narrow peak is observed as the individual vibrational mode of the amide carbonyl. The narrow nature of the Lorentzian peak suggests that most of compound **3** precipitates out of solution after sample preparation.^60^ Time-dependent FTIR spectra were recorded (Section S2.7) to further study the stability of the compound **3** dispersions. As a first order reaction, the peak intensity of FTIR was fitted to an exponential function to extract the associated time constants. This provided a time constant of compound **3** precipitation of 40 min (Figure S29). This result agrees with the visual assessment and the turbidimetry analysis experiments.

To confirm the peak assignments, vibrational analysis based on electronic structure calculations was performed at the BP86/SDD level of theory,^61−63^ using isolated MO **1**, thioester **2**, and amide **3** lipids. As shown in Figure 4A, the calculated vibrational spectra (gray) in the frequency region from 1550 to 1800 cm^–1^ are composed of two modes; a strong one (blue triangle) corresponding to the carbonyl stretching and a weak one (red triangle) corresponding to the C=C double bond of the lipid alkyl tail. The relative frequency and oscillator strength of these peaks in the calculated spectra match qualitatively with experiments and show that the C=C in MO lipids overlaps with the ester carbonyl, but the oscillator strength is much lower compared to the C=O; thus, the band can be interpreted as consisting solely of C=O stretching modes. These results show that the position and the strength of the calculated spectra match well with the experiments, suggesting that we have correct experimental peak assignments.

Finally, we aimed to corroborate our Laurdan, turbidimetry, and SAXS analyses of the phase transitions of each compound. Thus, we employed FTIR to understand how the interfacial environment of each compound changed in response to temperature. In Figure 4B, spectra measured at temperatures from 5 to 50 °C are shown for **1**, **2**, and **3**. In MO **1**, the 0HB/1HB ratio increases with increasing temperature, which results from the population changes associated with hydrogen bond formation and dissociation to the carbonyl.^64^ The spectra line shape of thioester **2** does not show a dependence on temperature, suggesting increased thermostability compared to MO **1**. In the amide **3** system, the ratio of 0HB/1HB peak decreases at higher temperatures, and significant 0HB peak broadening is observed. This indicates the decreasing insoluble aggregates and the presence of dispersed MO analogues in solution, showing that the solubility of amide **3** changes significantly above 45 °C. The spectra in the CH_2_ symmetric stretch region (Figure S30) also confirm that the MO **1** remains in the same phase throughout the temperature range, while in dispersions of the lipid **3** a significant change occurs at 45 °C, as observed by the different packing order of the acyl chains.^65^

## Conclusion

We have examined the self-assembly and temperature-dependent stability of MO analogues containing a thioester or amide, rather than an ester, linkage between the hydrophilic headgroup and hydrophobic tail of the lipid. Phase contrast microscopy and cryo-EM revealed that the large-scale structures of the thioester-containing compound **2** suggest a hexagonal phase arrangement, rather than the cubic phase structural arrangement of MO **1**. Alternatively, the amide-containing compound **3** formed vesicles which was validated by the presence of the lamellar phase determined via SAXS. By integrating our Laurdan GP, turbidimetry, and FTIR results, we have shown that although MO **1** forms a stable dispersion at all temperatures ranging from 5 to 50 °C, compounds **2** and **3** have decreased stability at lower temperatures, and dramatic structural changes occur at around 45 °C for dispersions of compound **3**. It is worth noting that the striking differences in the particle structures may partially be determined by the formation of H-bonding interactions between lipids. This interaction is possible for amide **3** which contains both an H-bond acceptor and donor, while it cannot occur for MO **1** and thioester **2**. Furthermore, substituting the oxygen (compound **1**) with sulfur (compound **2**) can affect the hydrogen bonding with water at the interface because the thioester is a better H-bond acceptor. Our results suggest that strong directional intermolecular interactions among the linking functional group near the headgroup region play a key role in determining the intermolecular interactions between self-assembling amphiphiles and at the amphiphile–water interface. Subsequently, these molecular interactions define the nature of the large-scale assemblies formed by each MO analogue. The observation that the linking functional group of MO drastically influences the self-assembly properties of lipid mesophases suggests there are many additional avenues to explore the properties of MO isosteres. We envision future work will fine-tune the characteristics of additional MO isosteres to develop mesophases with distinct physical properties and assemblies. These properties could introduce new functionalities into artificial cell compartments or find application as novel nanomaterials for nanomedicine.

## Supporting Information Available

NMR spectral images, Scheme S1 (synthesis of compound **2**), Scheme S2 (synthesis of compound **3**), Figures S5 and S6 (light microscopy images of compounds **2** and **3**), Figures S7 and S8 (cryo-EM images of compounds **2** and **3**), Figure S9 (SAXS of compound **3**), Figures S10–S12 (fluorescence spectra of Laurdan in lipid dispersions of compounds **1**, **2**, and **3**), Figures S13–S28 (stability of lipid mesophases by turbidimetry analysis), and Figures S29 and S30 (temperature-dependent FTIR spectra) (PDF)
