# Supplementary material for: Characterizing the Self-Assembly Properties of Monoolein Lipid Isosteres
Source: J Phys Chem B. 2023 Feb 16;127(8):1771–9. doi: 10.1021/acs.jpcb.2c07215 (PMC9986874; doi:10.1021/acs.jpcb.2c07215)
Supplement: Supplementary file 1 — jp2c07215_si_001.pdf [file jp2c07215_si_001.pdf]

Characterizing the Self-assembly Properties of Monoolein Lipid Isosteres

Alessandro Fracassi,+[a] Kira A. Podolsky,[a] Sudip Pandey,[b] Cong Xu,[c] Joshua Hutchings,[d] Soenke Seifert,[e] Carlos R. Baiz,[c] Sunil K. Sinha,[b] and Neal K. Devaraj*[a]

[a] Department of Chemistry and Biochemistry, University of California, San Diego, 9500 Gilman Drive, Natural Sciences Building 3328, CA 92093, USA.

[b] Department of Physics, University of California, San Diego, 9500 Gilman Drive, Mayer Hall Addition 4561, CA 92093, USA.

[c] Department of Chemistry, The University of Texas at Austin, 105 E. 24th St. Stop A5300, Austin, TX 78712–1224, USA.

[d] Department of Molecular Biology, School of Biological Sciences, University of California, San Diego, La Jolla, CA 92093, USA.

[e] X-ray Science Division, Argonne National Laboratory, 9700 South Cass Avenue, Argonne, IL 60439, USA.

Corresponding author: Neal K. Devaraj
Email: ndevaraj@ucsd.edu

Supporting Information

Table of Contents

1. Synthesis of monoolein analogs 3

1.1 Synthesis of thioester 2[1] 3

1.2 Synthesis of amide 3[1] 4

2. Lipid mixtures characterization 6

2.1 Light microscopy 6

2.2 Cryo-electron microscopy (cryoEM) 9

2.3 Small angle X-ray scattering (SAXS) 11

2.4 Fluorescence spectra of Laurdan in monoolein analogs dispersion 12

2.5 Stability of lipid mesophases by turbidimetry analysis 14

2.6 Visual assessment of lipid dispersion stability 26

2.7 Fourier-transform IR (FTIR) spectroscopy 30

2.8 Electronic structure models 31

3. References 32

1. Synthesis of monoolein analogs

1.1 Synthesis of thioester 2[1]

Scheme S1. Synthesis of thioester **2**

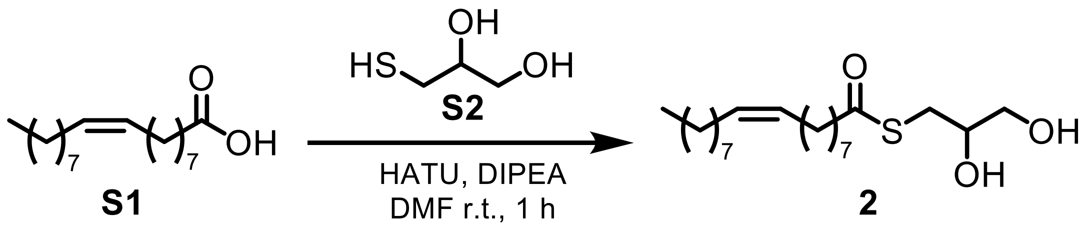

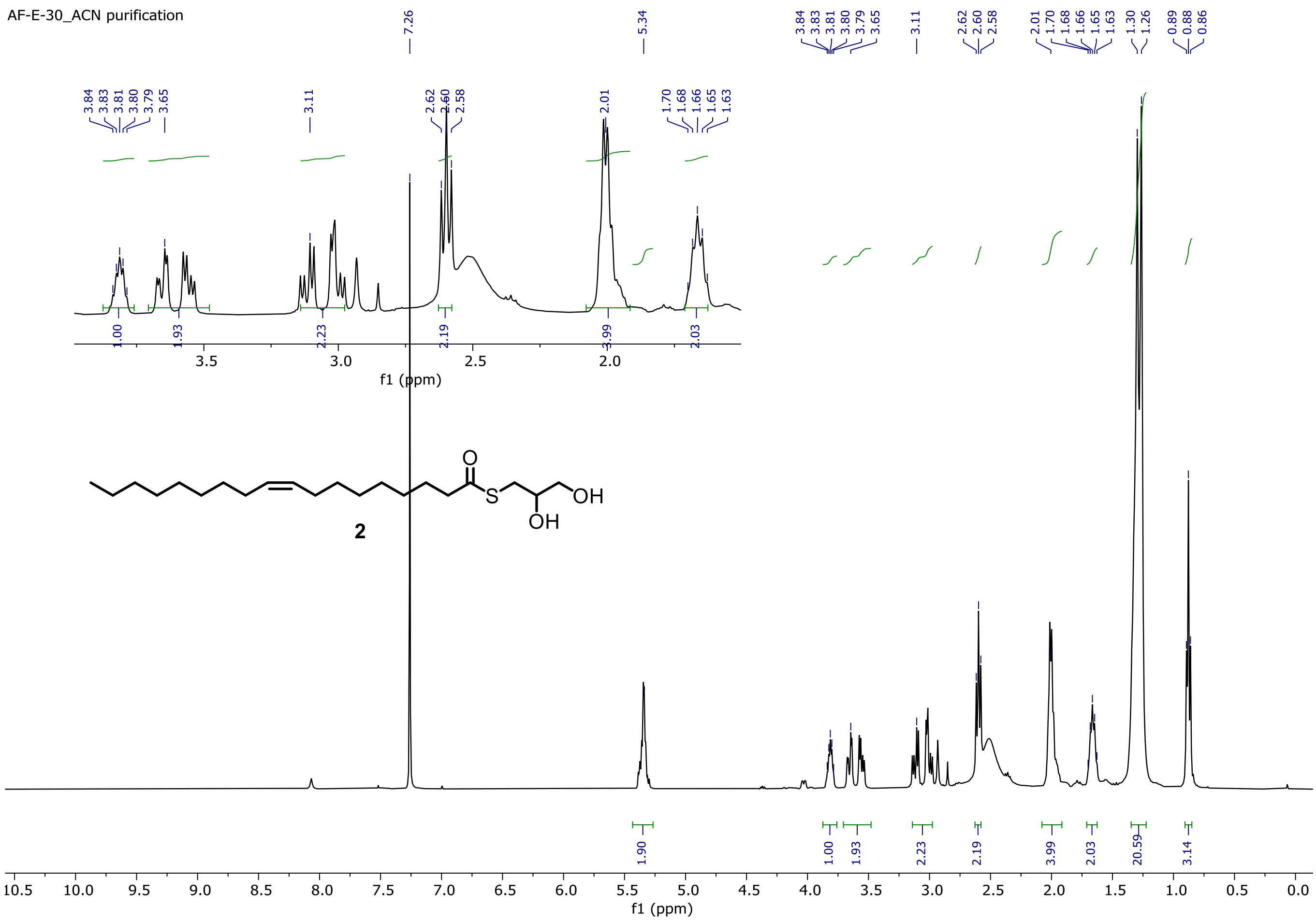

Figure S1. ^{1}H NMR spectrum of **2** (in $CDCl_3$, 500 MHz).

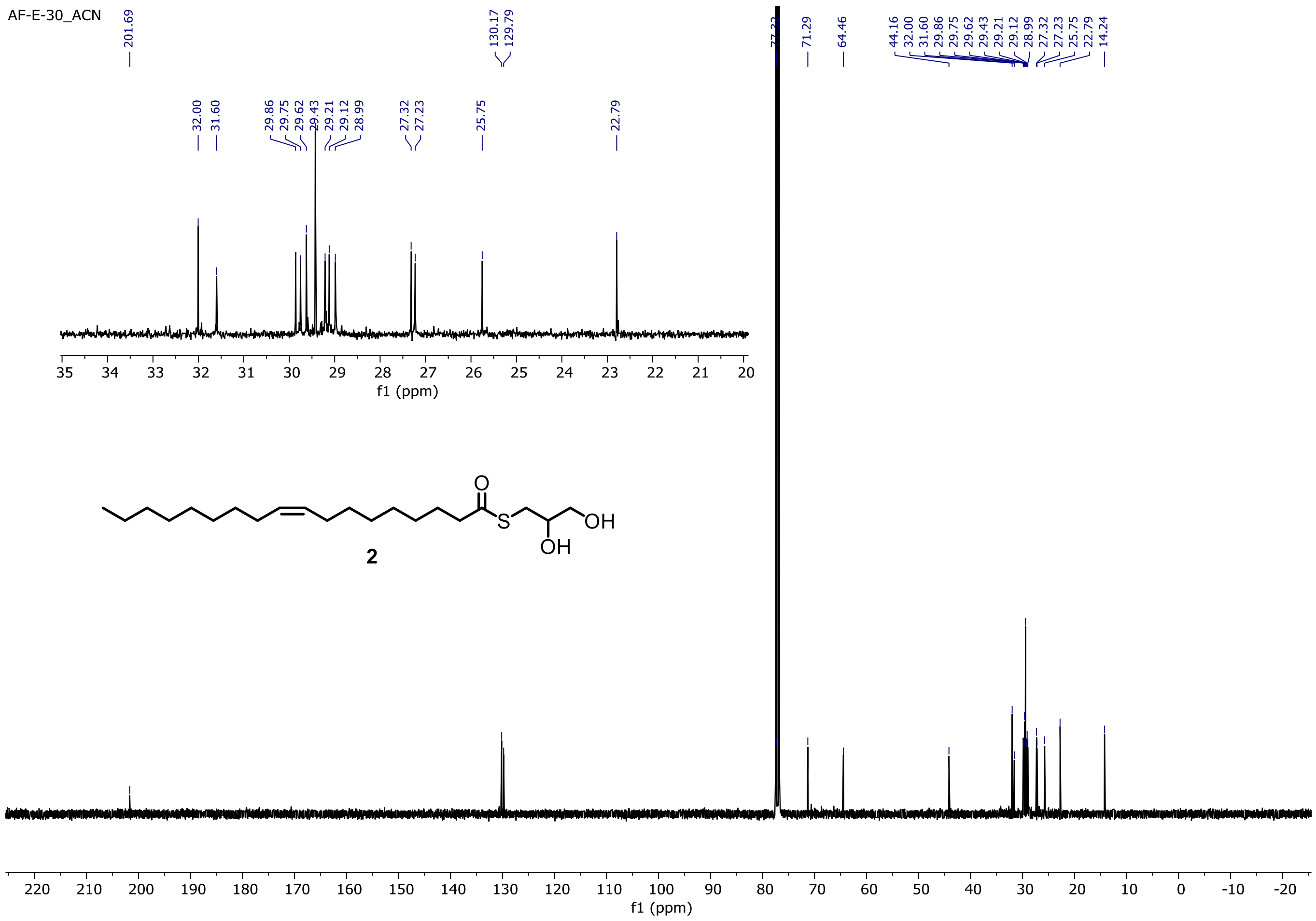

Figure S2. ^{13}C NMR spectrum of **2** (in $CDCl_3$, 125 MHz).

1.2 Synthesis of amide 3[1]

Scheme S2. Synthesis of amide **3**

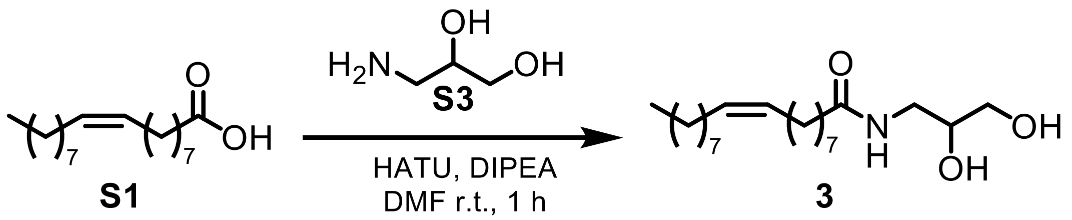

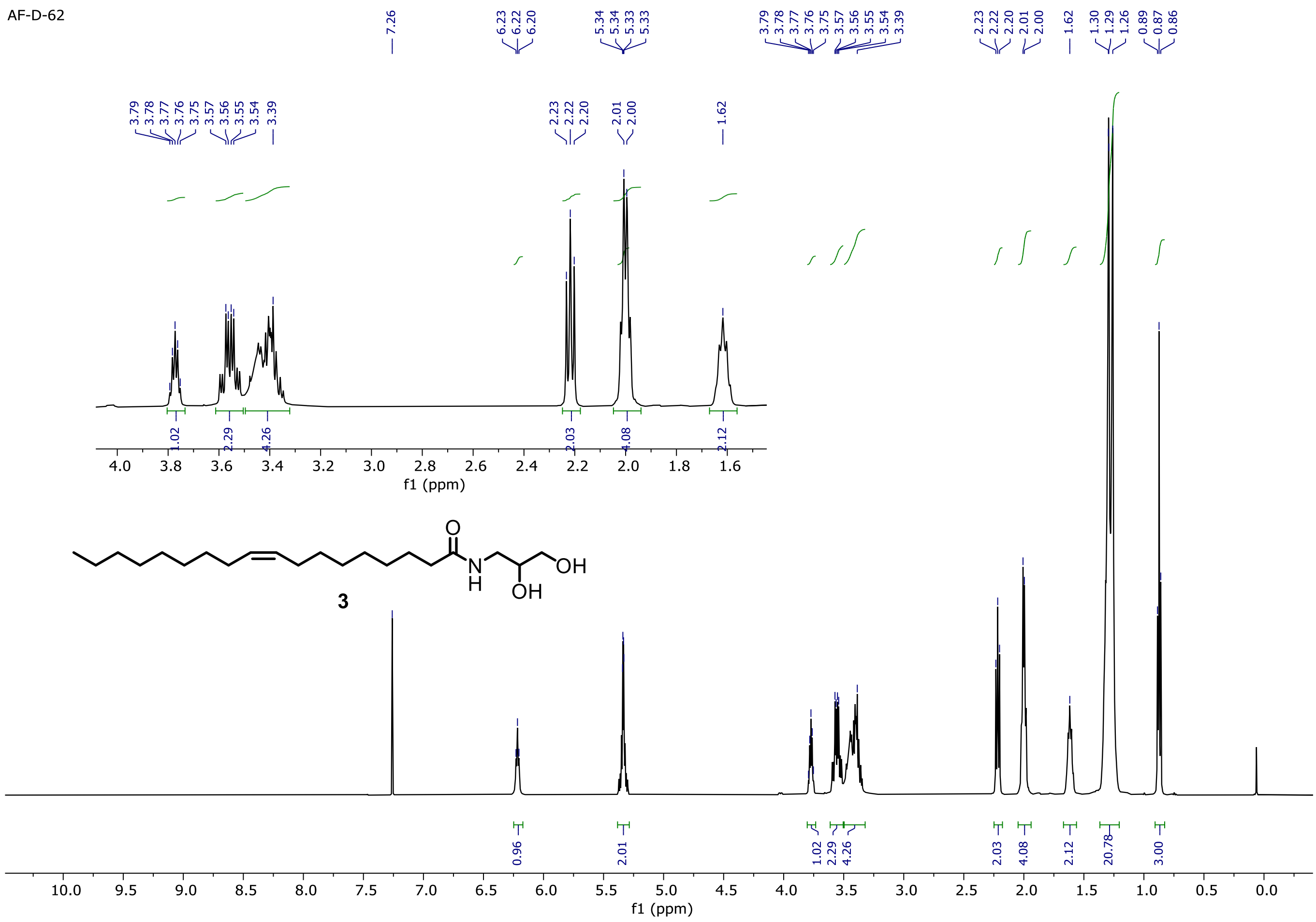

Figure S3. ^{1}H NMR spectrum of **3** (in $CDCl_3$, 500 MHz).

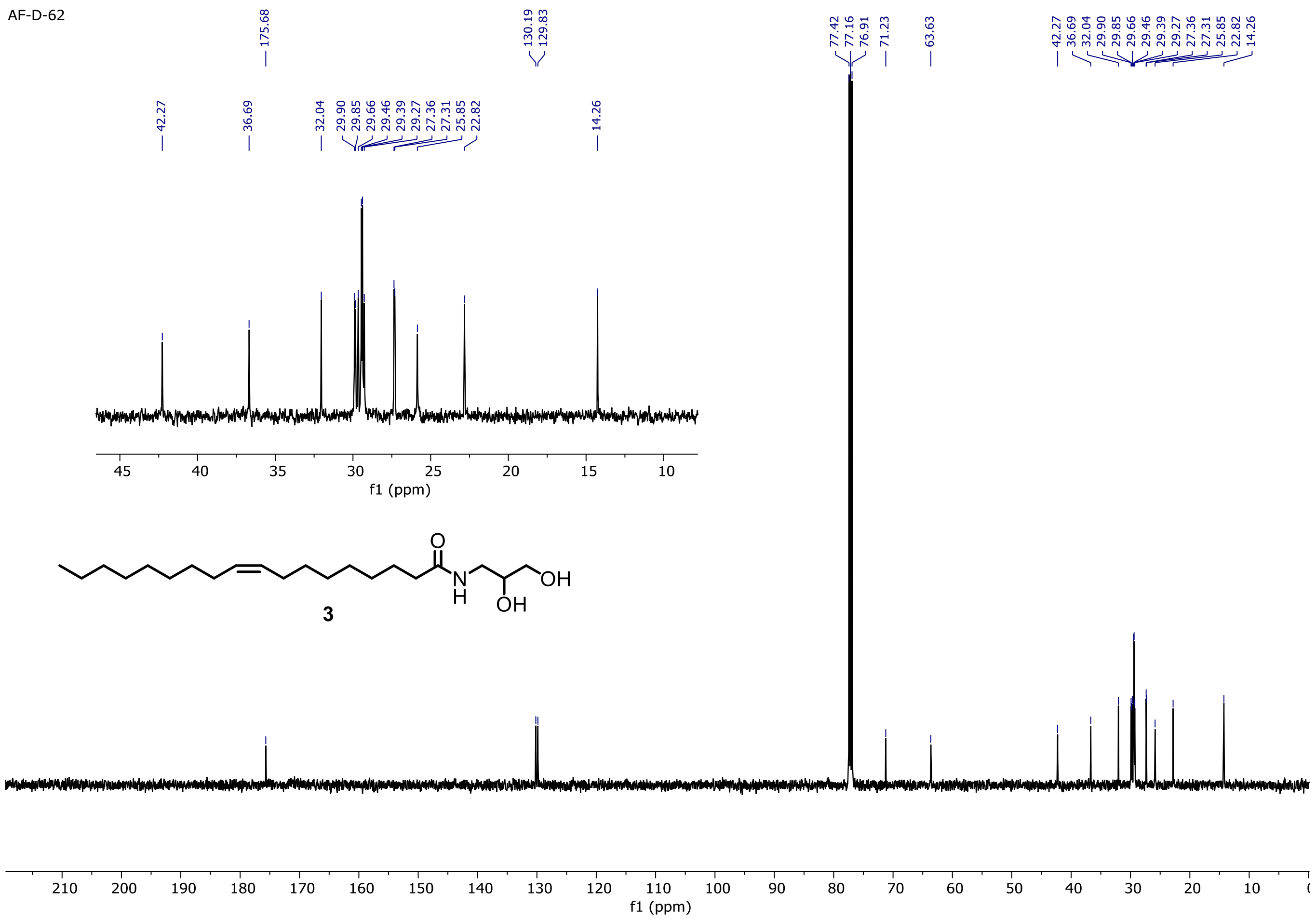

Figure S4. ^{13}C NMR spectrum of **3** (in $CDCl_3$, 125 MHz).

2. Lipid mixtures characterization

2.1 Light microscopy

Microscopy images were acquired using an Olympus BX51 optical microscope. Images were further analyzed with ImageJ software (version 2.1.0). The samples were prepared by depositing 2 µL of the desired lipid dispersion on a microscope glass slide (Fisher brand), and topping with a coverslip.

Sample A

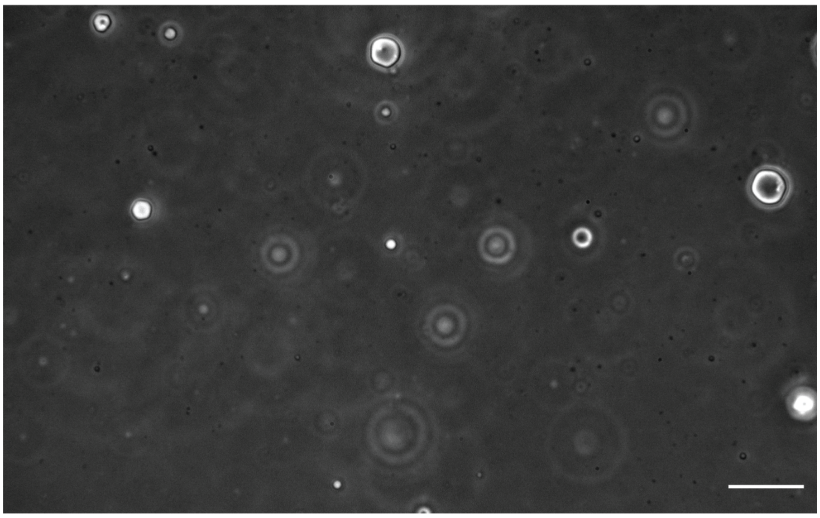

Sample B

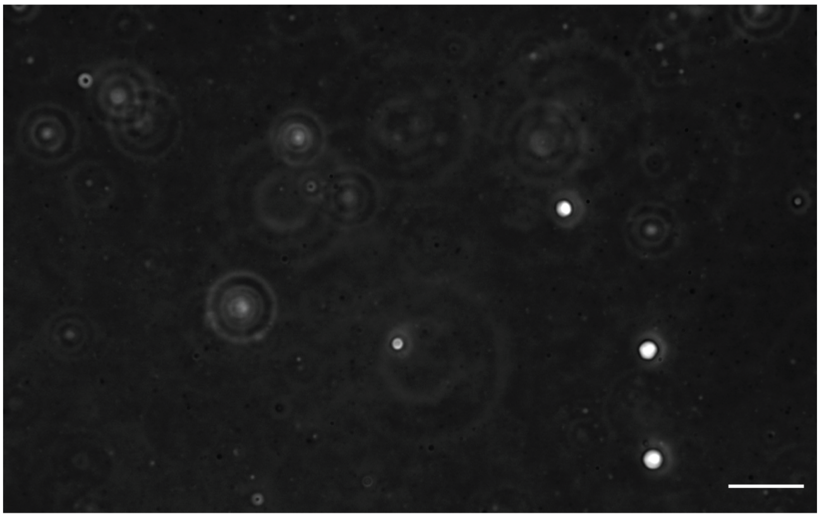

Sample C

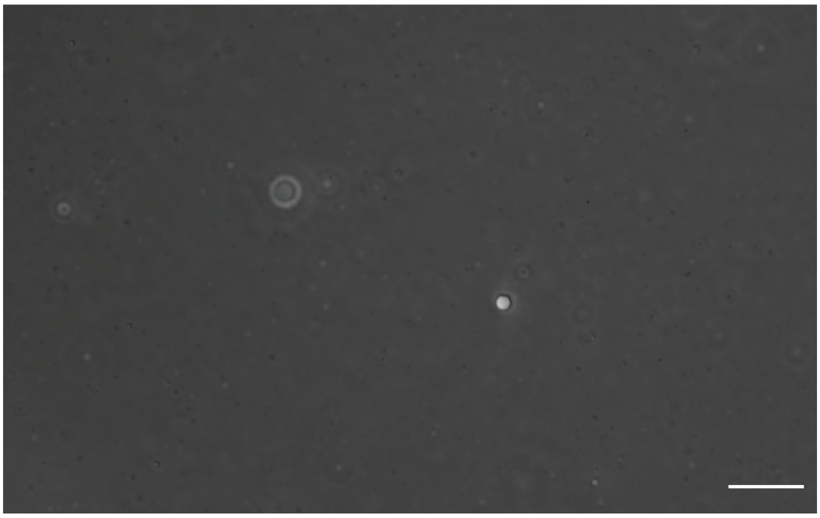

Figure S5. Phase contrast microscopy images at 25 °C of three different samples of a 10 mM dispersion of compound 2 in water containing Pluronic F127 (15 μM). Scale bars denote 25 μm.

Sample A

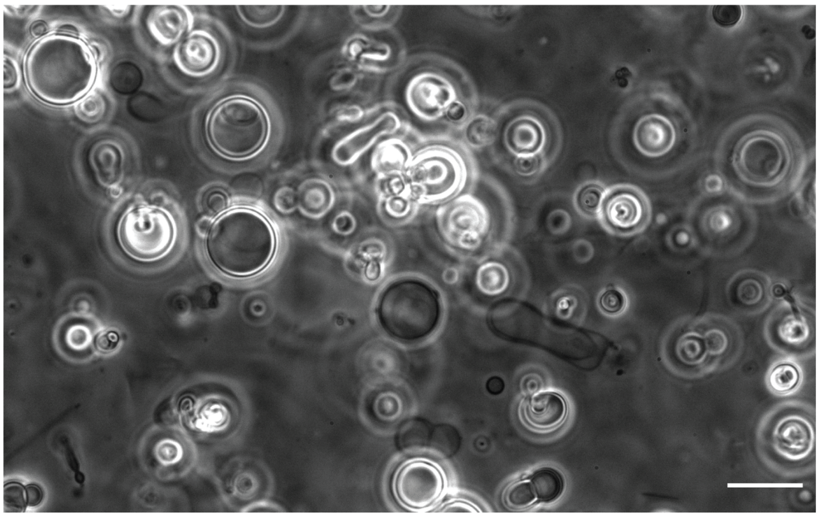

Sample B

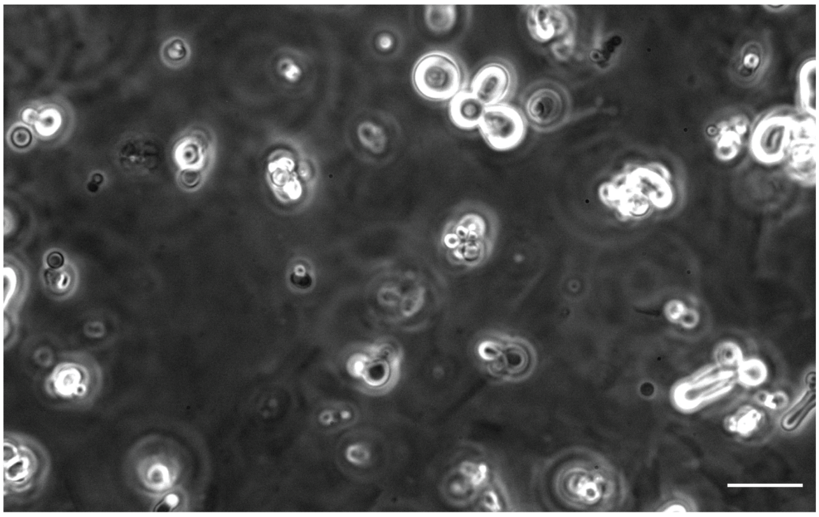

Sample C

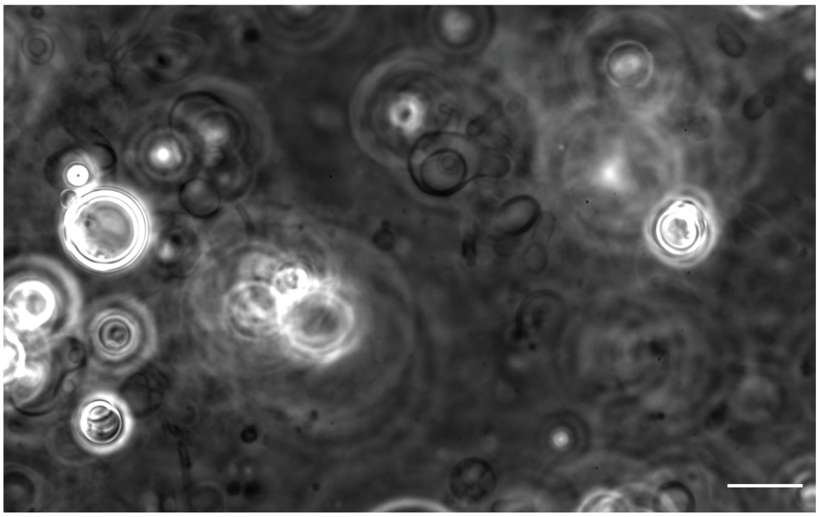

Figure S6. Phase contrast microscopy images at 25 °C of three different samples of a 10 mM dispersion of compound **3** in water containing Pluronic F127 (15 μM). Scale bars denote 25 μm.

2.2 Cryo-electron microscopy (cryoEM)

Image A

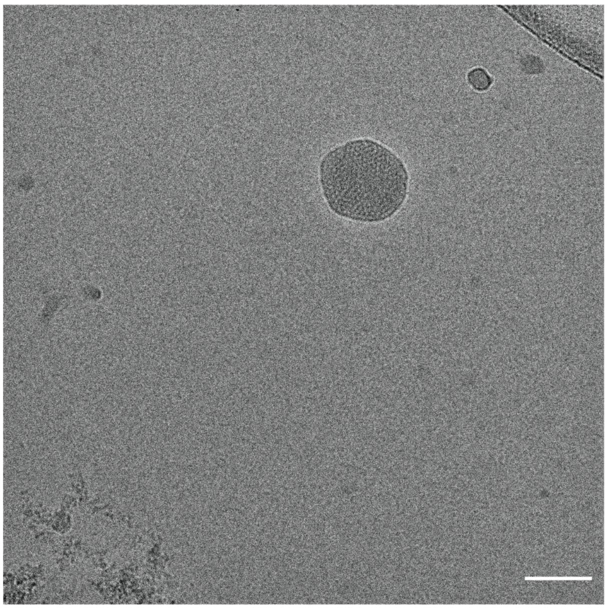

Image B

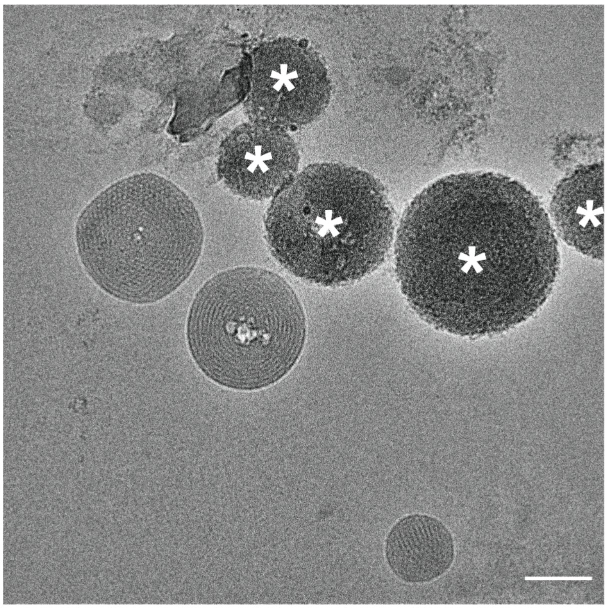

Figure S7. Cryo-EM micrographs of a 10 mM dispersion of compound **2** in water containing Pluronic F127 (15 μM). Asterisks denote contamination in the form of frozen particles not embedded within the vitrified ice. Scale bar denotes 100 nm.

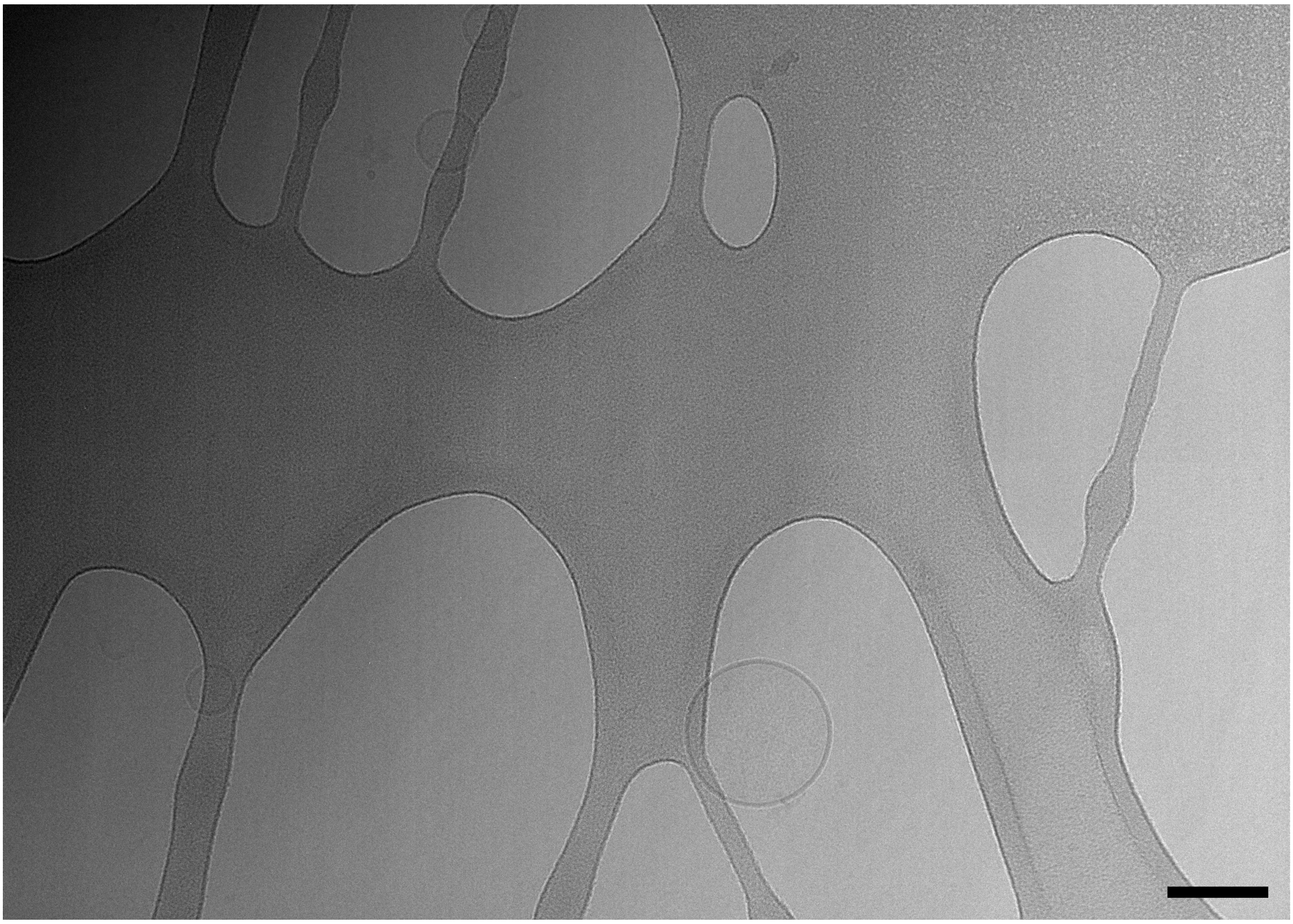

Figure S8. Cryo-EM micrographs of a 10 mM dispersion of compound **3** in water containing Pluronic F127 (15 μM). Scale bar denotes 100 nm.

2.3 Small angle X-ray scattering (SAXS)

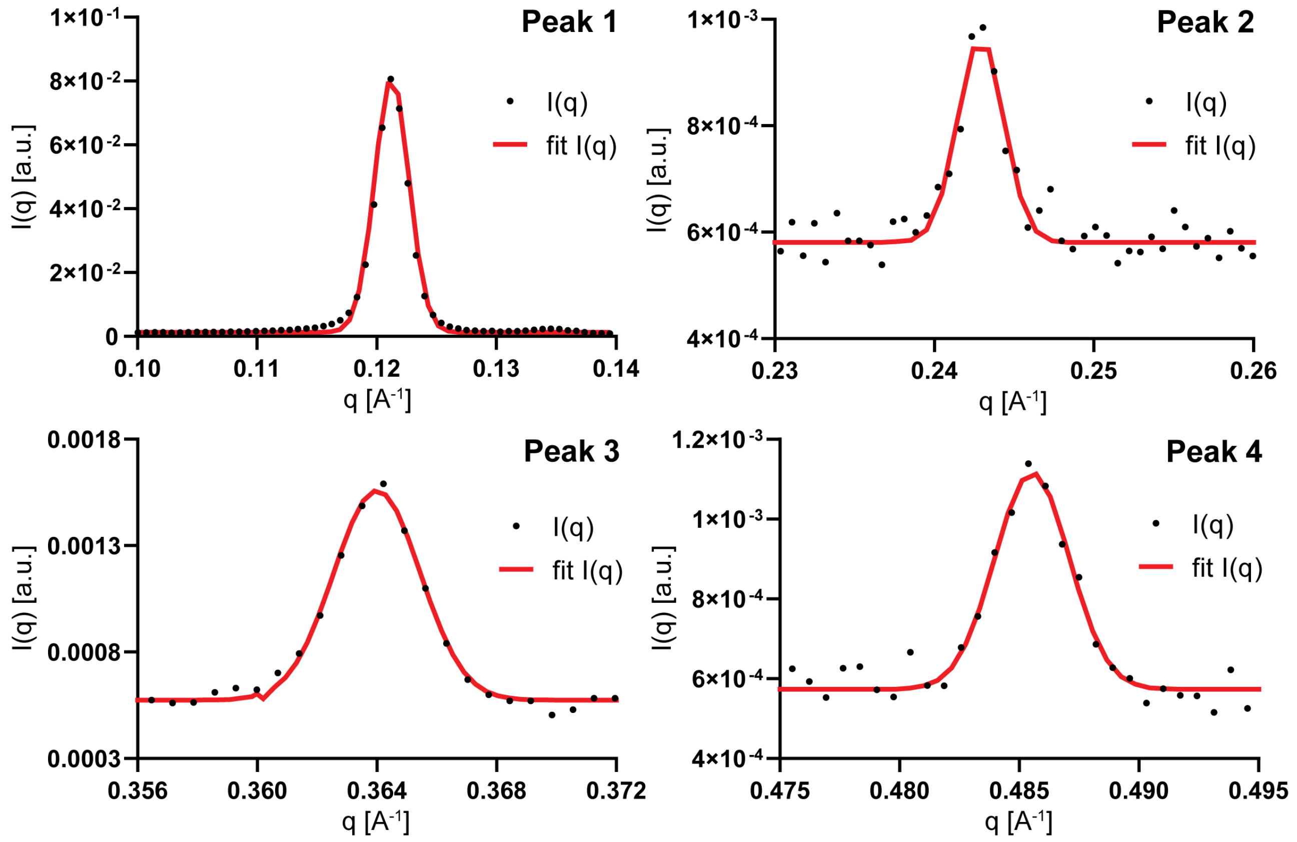

Figure S9. Synchrotron SAXS intensity profile (dotted black) recorded at 25 °C with fitting (solid red) of a lipid dispersion of compound **3**, showing the presence of four distinct peaks.

2.4 Fluorescence spectra of Laurdan in monoolein analogs dispersion

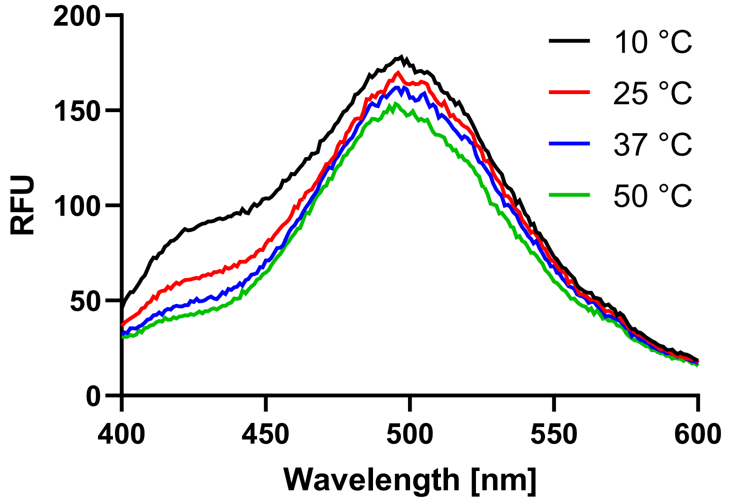

Figure S10. Fluorescence spectra of Laurdan in a dispersion of compound **1** (10 mM) at 10, 25, 37, 50 °C. The fluorescence spectra showed a maximum emission at 490 nm (λ_{ex} = 360 nm).

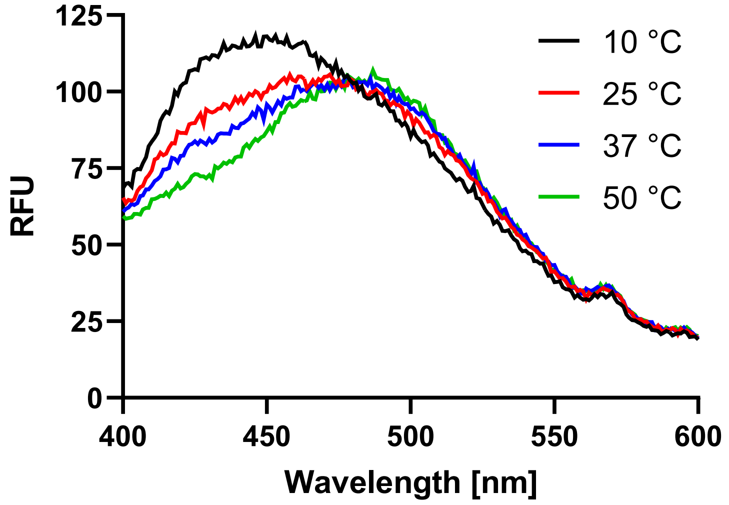

Figure S11 Fluorescence spectra of Laurdan in a dispersion of compound **2** (10 mM) at 10, 25, 37, 50 °C. The fluorescence spectrum at 10 °C showed a maximum emission at 440 nm (λ_{ex} = 360 nm), which shifted to 465 nm at 25 °C, and to 490 nm at 37, and 50 °C.

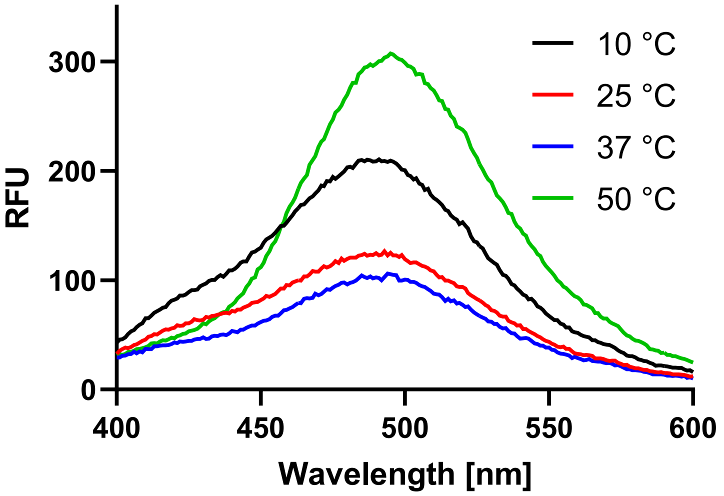

Figure S12. Fluorescence spectra of Laurdan in a dispersion of compound **3** (10 mM) at 10, 25, 37, 50 °C. The fluorescence spectra showed a maximum emission at 490 nm (λ_{ex} = 360 nm).

2.5 Stability of lipid mesophases by turbidimetry analysis

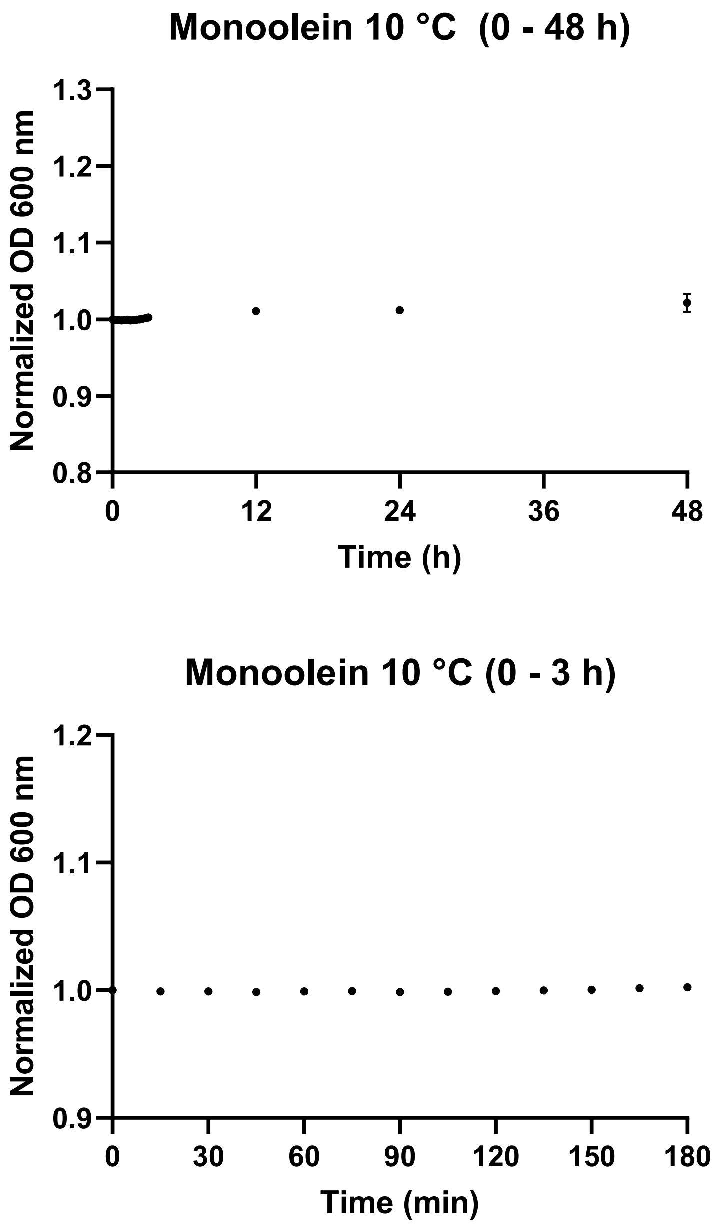

Figure S13. Turbidimetry measurements of a dispersion of compound **1** in water containing Pluronic F127 (15 μM) recorded at 10 °C monitoring the optical density at 600 nm (OD_{600}). The *top* figure shows the normalized OD_{600} obtained by measuring every 15 min over 3 h, and at 12, 24, and 48 h. The *bottom* figure shows the zoom of the first 3 h measurements. The figure shows the average of three measurements with the error bars indicating the standard deviation.

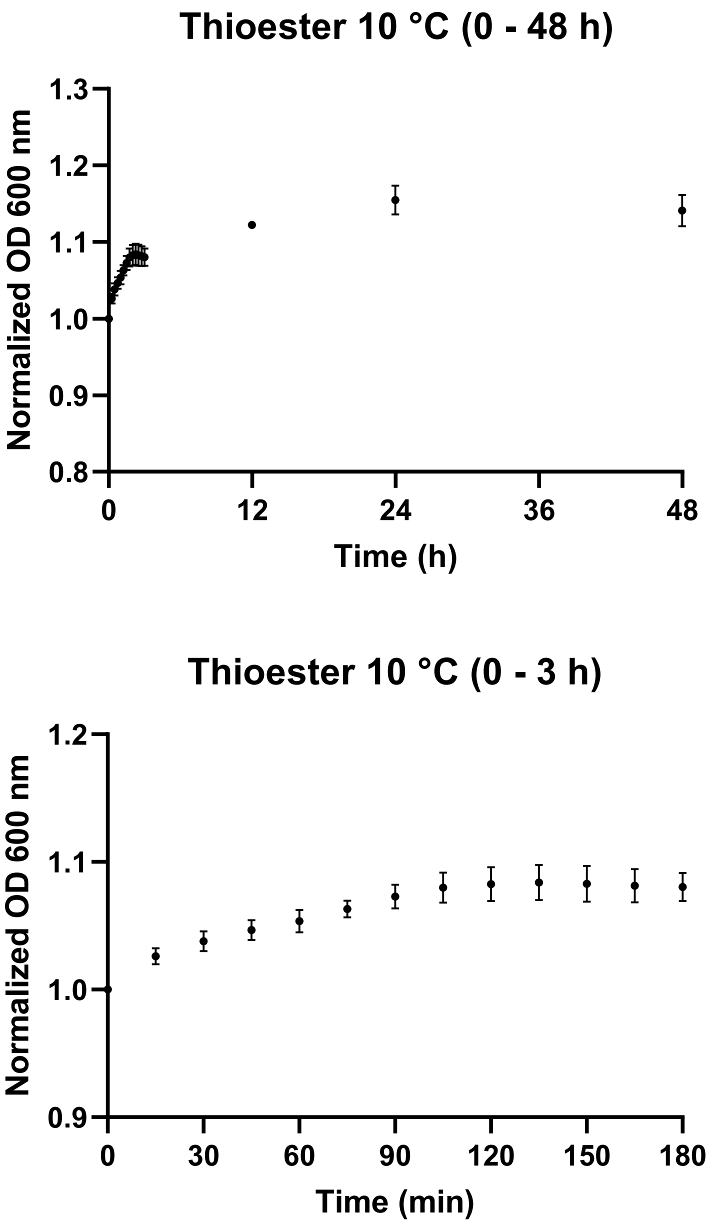

Figure S14. Turbidimetry measurements of a dispersion of compound **2** in water containing Pluronic F127 (15 μM) recorded at 10 °C monitoring the optical density at 600 nm (OD_{600}). The *top* figure shows the normalized OD_{600} obtained by measuring every 15 min over 3 h, and at 12, 24, and 48 h. The *bottom* figure shows the zoom of the first 3 h measurements. The figure shows the average of three measurements with the error bars indicating the standard deviation.

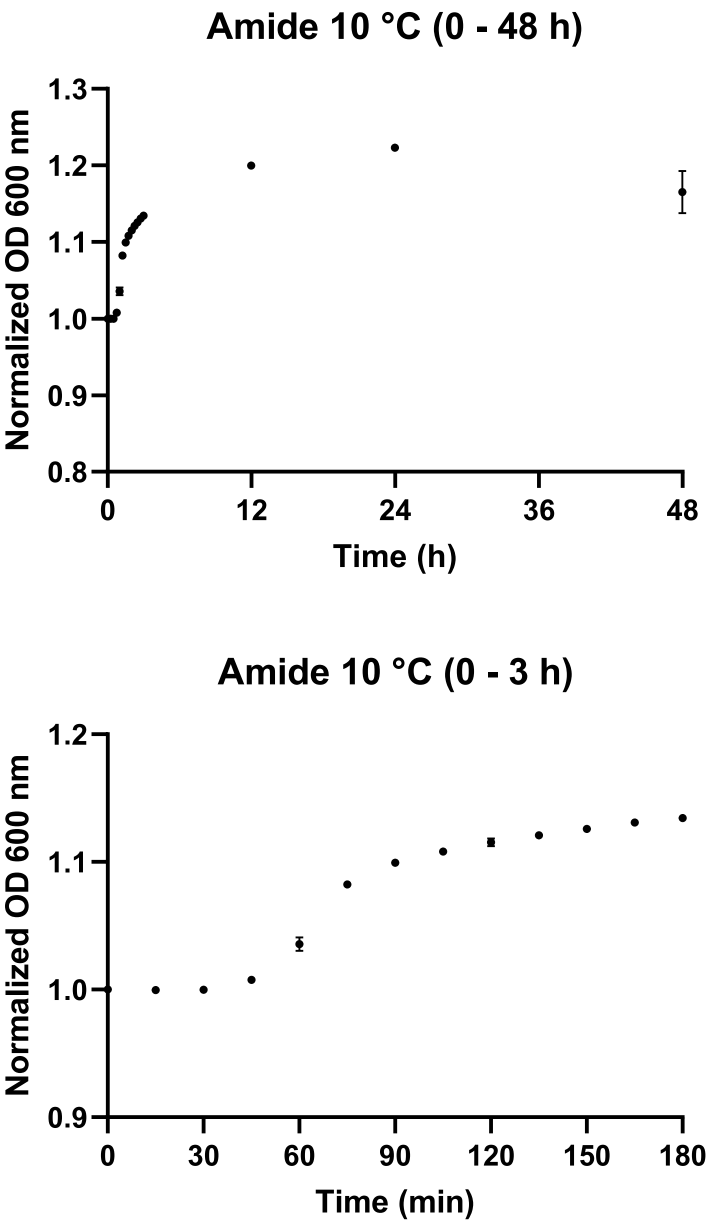

Figure S15. Turbidimetry measurements of a dispersion of compound **3** in water containing Pluronic F127 (15 μM) recorded at 10 °C monitoring the optical density at 600 nm (OD_{600}). The *top* figure shows the normalized OD_{600} obtained by measuring every 15 min over 3 h, and at 12, 24, and 48 h. The *bottom* figure shows the zoom of the first 3 h measurements. The figure shows the average of three measurements with the error bars indicating the standard deviation.

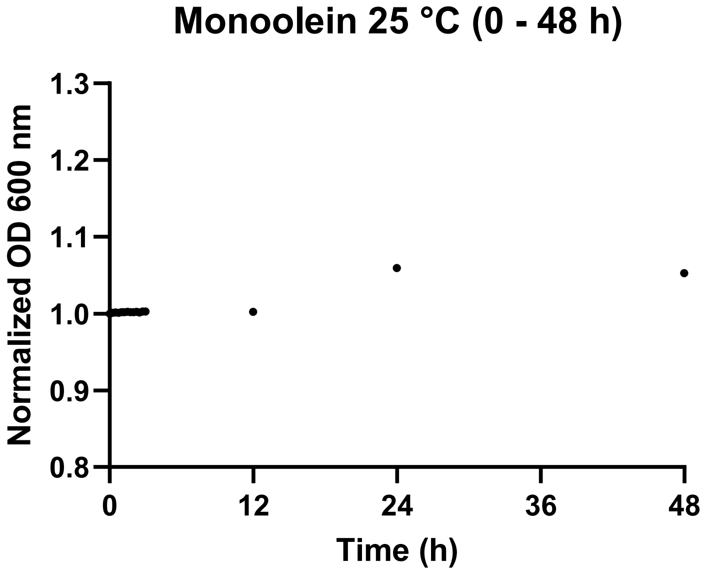

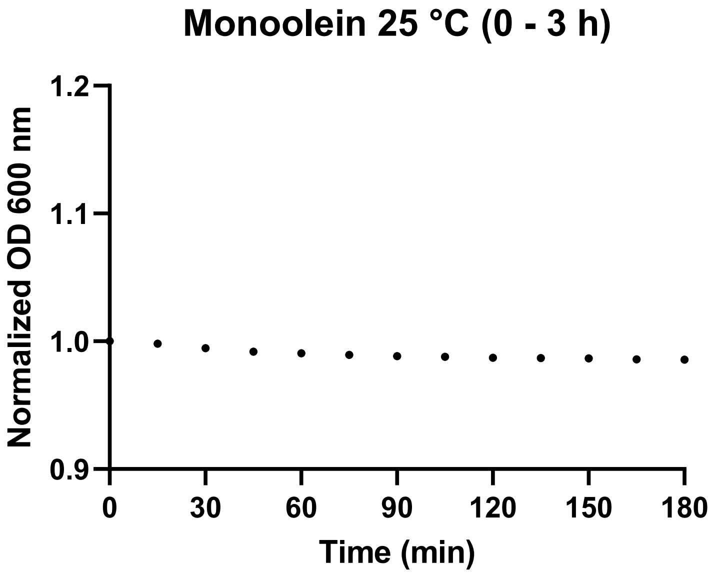

Figure S16. Turbidimetry measurements of a dispersion of compound **1** in water containing Pluronic F127 (15 μM) recorded at 25 °C monitoring the optical density at 600 nm (OD_{600}). The *top* figure shows the normalized OD_{600} obtained by measuring every 15 min over 3 h, and at 12, 24, and 48 h. The *bottom* figure shows the zoom of the first 3 h measurements. The figure shows the average of three measurements with the error bars indicating the standard deviation.

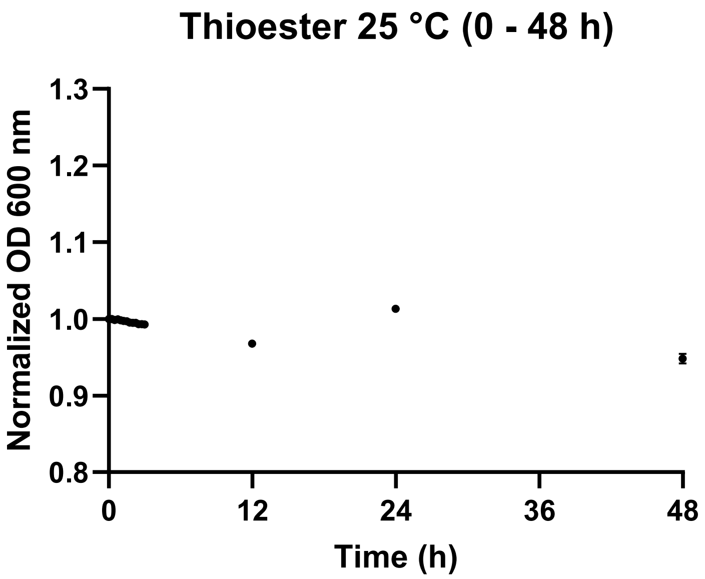

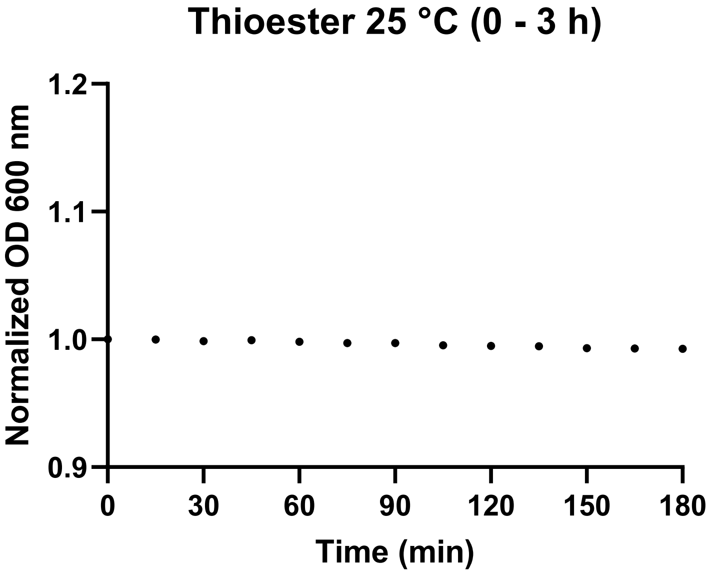

Figure S17. Turbidimetry measurements of a dispersion of compound **2** in water containing Pluronic F127 (15 μM) recorded at 25 °C monitoring the optical density at 600 nm (OD_{600}). The *top* figure shows the normalized OD_{600} obtained by measuring every 15 min over 3 h, and at 12, 24, and 48 h. The *bottom* figure shows the zoom of the first 3 h measurements. The figure shows the average of three measurements with the error bars indicating the standard deviation.

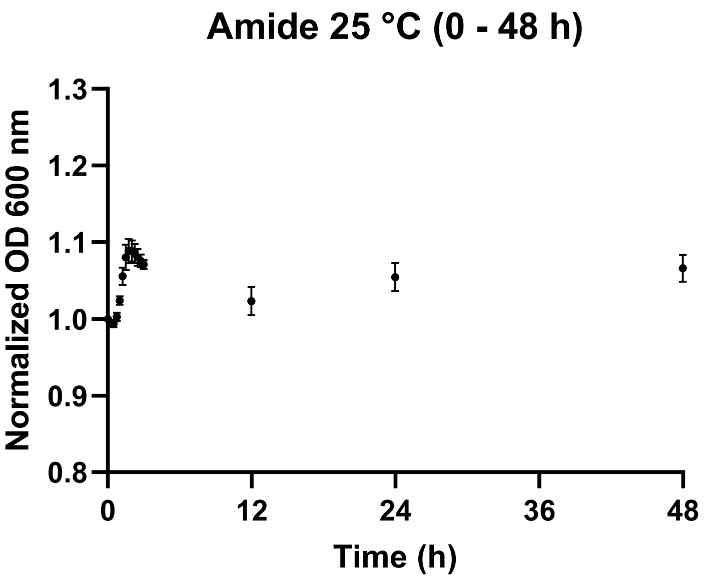

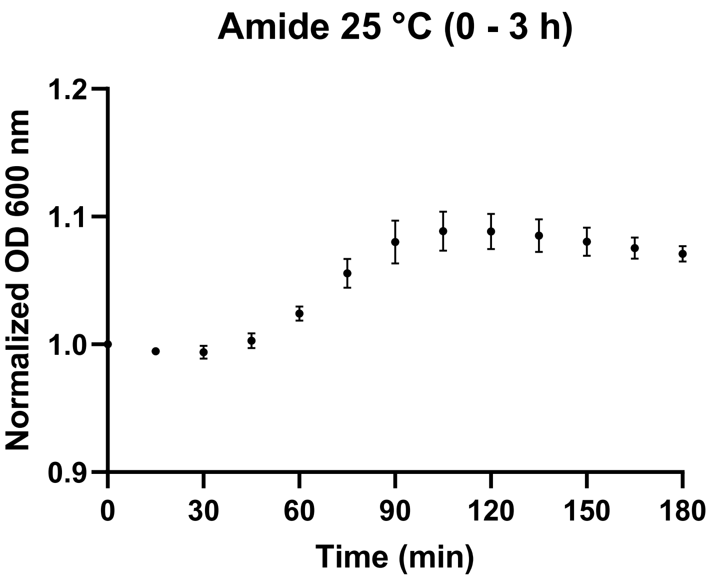

Figure S18. Turbidimetry measurements of a dispersion of compound **3** in water containing Pluronic F127 (15 μM) recorded at 25 °C monitoring the optical density at 600 nm (OD_{600}). The *top* figure shows the normalized OD_{600} obtained by measuring every 15 min over 3 h, and at 12, 24, and 48 h. The *bottom* figure shows the zoom of the first 3 h measurements. The figure shows the average of three measurements with the error bars indicating the standard deviation.

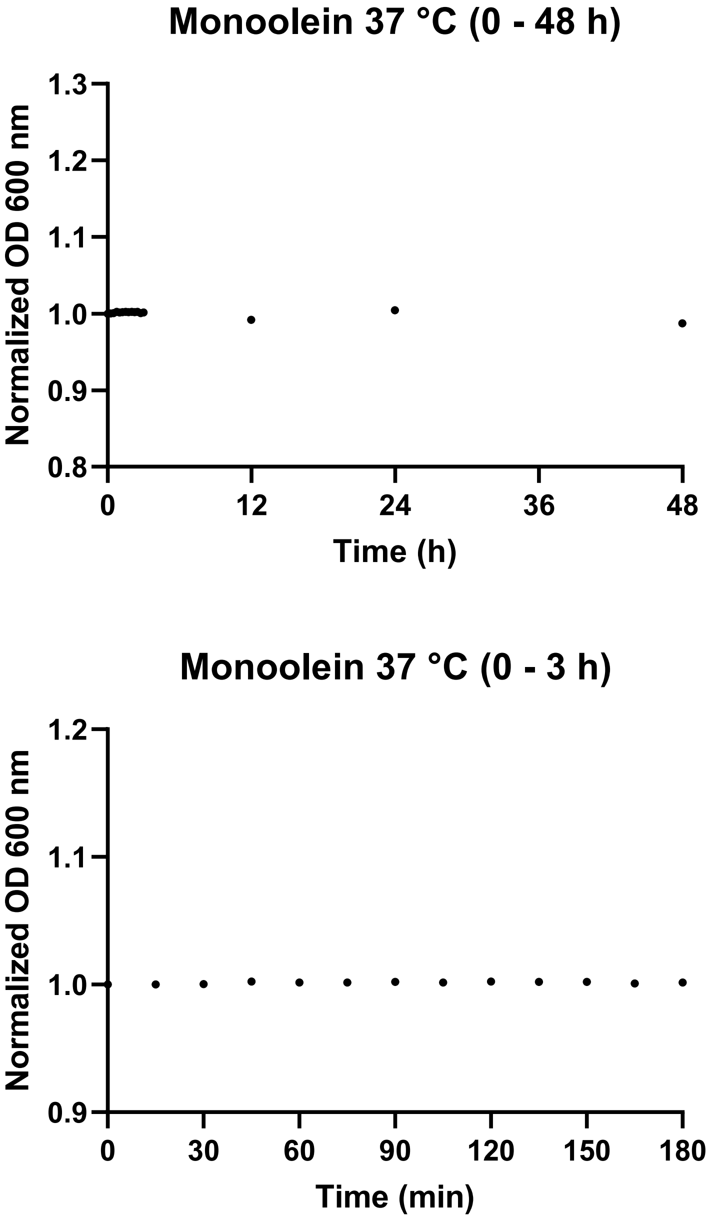

Figure S19. Turbidimetry measurements of a dispersion of compound **1** in water containing Pluronic F127 (15 µM) recorded at 37 °C monitoring the optical density at 600 nm (OD_{600}). The *top* figure shows the normalized OD_{600} obtained by measuring every 15 min over 3 h, and at 12, 24, and 48 h. The *bottom* figure shows the zoom of the first 3 h measurements. The figure shows the average of three measurements with the error bars indicating the standard deviation.

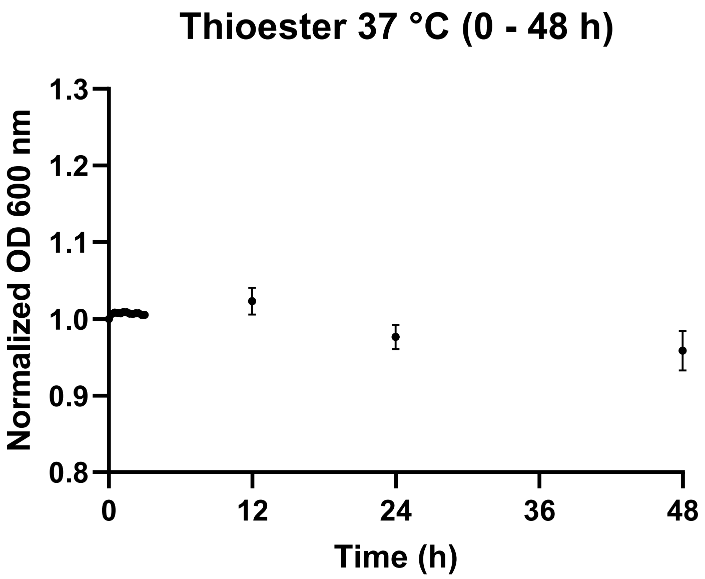

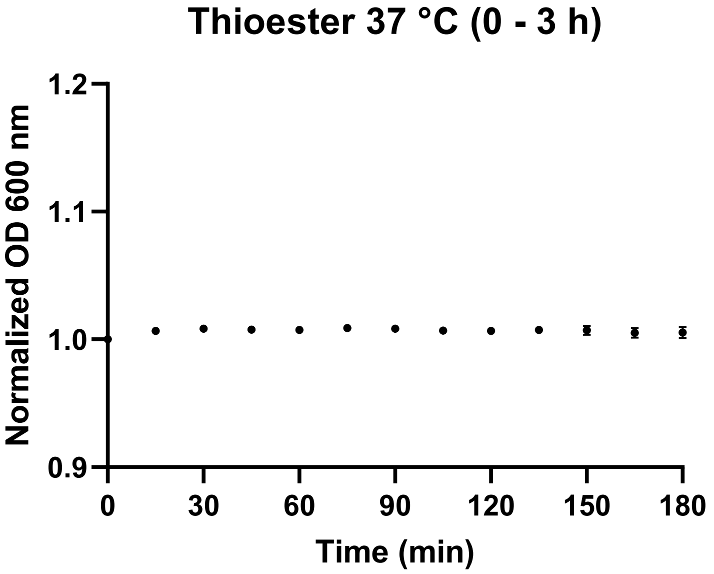

Figure S20. Turbidimetry measurements of a dispersion of compound **2** in water containing Pluronic F127 (15 µM) recorded at 37 °C monitoring the optical density at 600 nm (OD_{600}). The *top* figure shows the normalized OD_{600} obtained by measuring every 15 min over 3 h, and at 12, 24, and 48 h. The *bottom* figure shows the zoom of the first 3 h measurements. The figure shows the average of three measurements with the error bars indicating the standard deviation.

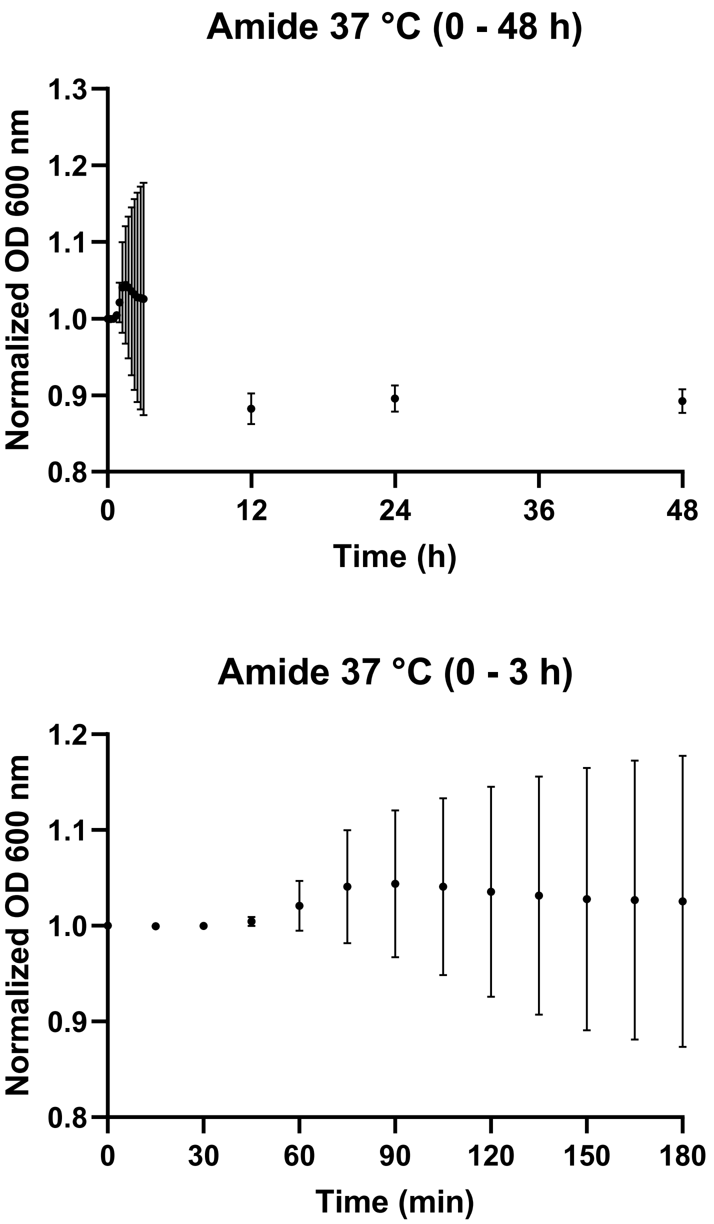

Figure S21. Turbidimetry measurements of a dispersion of compound **3** in water containing Pluronic F127 (15 µM) recorded at 37 °C monitoring the optical density at 600 nm (OD_{600}). The *top* figure shows the normalized OD_{600} obtained by measuring every 15 min over 3 h, and at 12, 24, and 48 h. The *bottom* figure shows the zoom of the first 3 h measurements. The figure shows the average of three measurements with the error bars indicating the standard deviation.

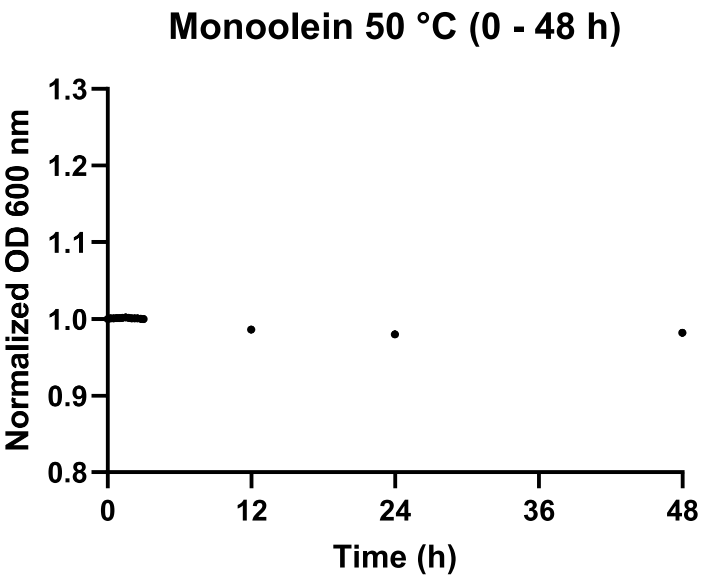

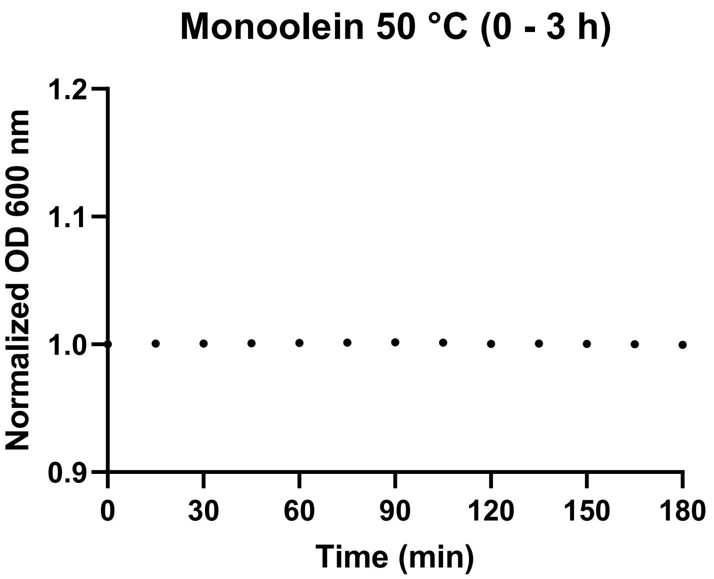

Figure S22. Turbidimetry measurements of a dispersion of compound **1** in water containing Pluronic F127 (15 µM) recorded at 50 °C monitoring the optical density at 600 nm (OD_{600}). The *top* figure shows the normalized OD_{600} obtained by measuring every 15 min over 3 h, and at 12, 24, and 48 h. The *bottom* figure shows the zoom of the first 3 h measurements. The figure shows the average of three measurements with the error bars indicating the standard deviation.

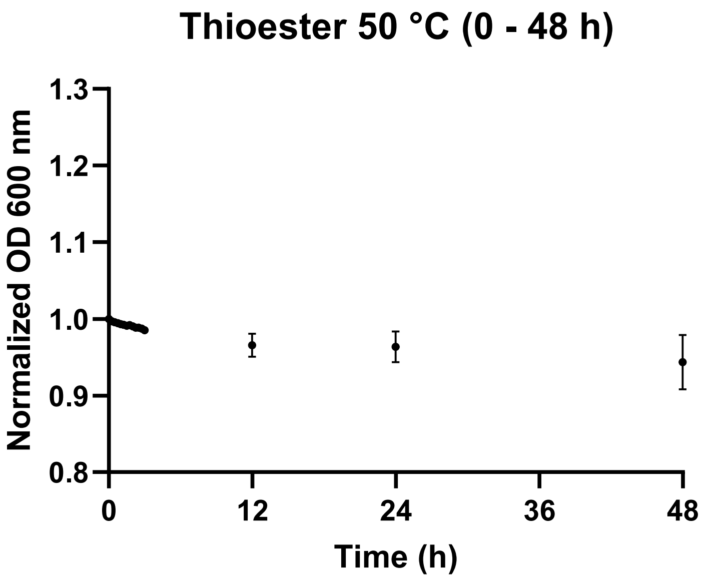

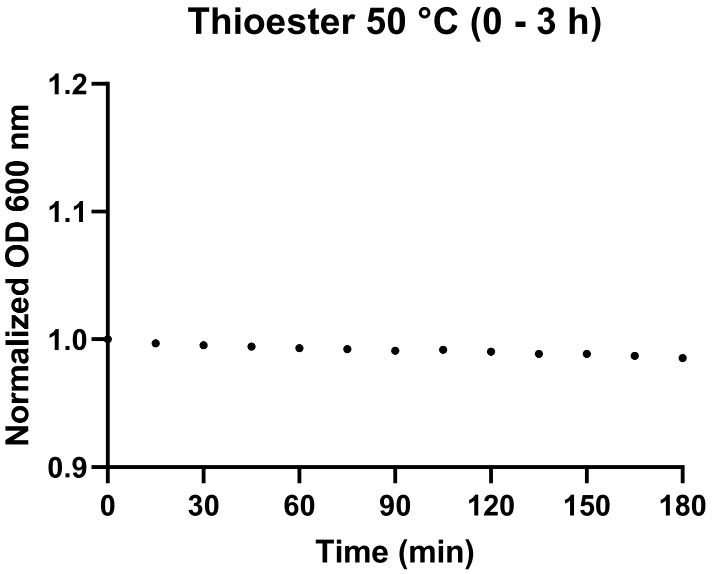

Figure S23. Turbidimetry measurements of a dispersion of compound **2** in water containing Pluronic F127 (15 µM) recorded at 50 °C monitoring the optical density at 600 nm (OD_{600}). The *top* figure shows the normalized OD_{600} obtained by measuring every 15 min over 3 h, and at 12, 24, and 48 h. The *bottom* figure shows the zoom of the first 3 h measurements. The figure shows the average of three measurements with the error bars indicating the standard deviation.

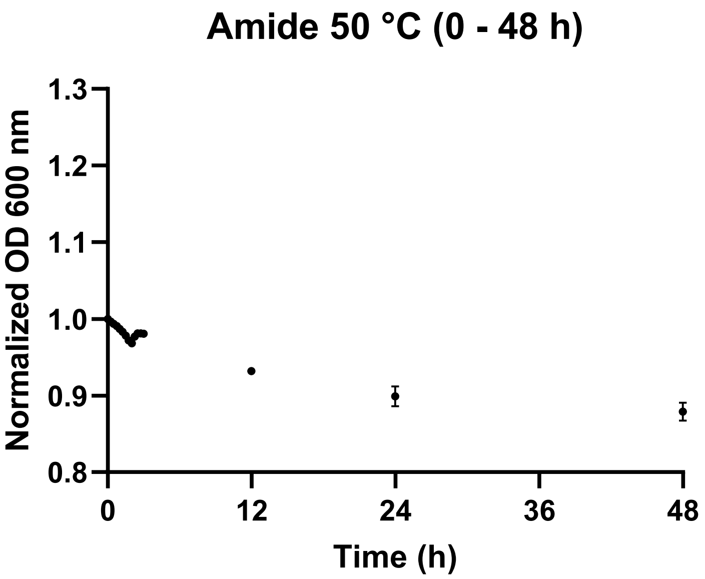

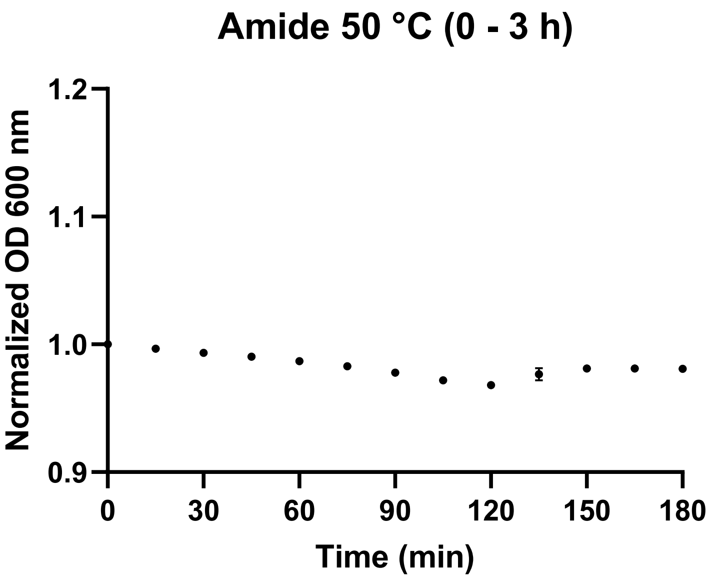

Figure S24. Turbidimetry measurements of a dispersion of compound **3** in water containing Pluronic F127 (15 µM) recorded at 50 °C monitoring the optical density at 600 nm (OD_{600}). The *top* figure shows the normalized OD_{600} obtained by measuring every 15 min over 3 h, and at 12, 24, and 48 h. The *bottom* figure shows the zoom of the first 3 h measurements. The figure shows the average of three measurements with the error bars indicating the standard deviation.

2.6 Visual assessment of lipid dispersion stability

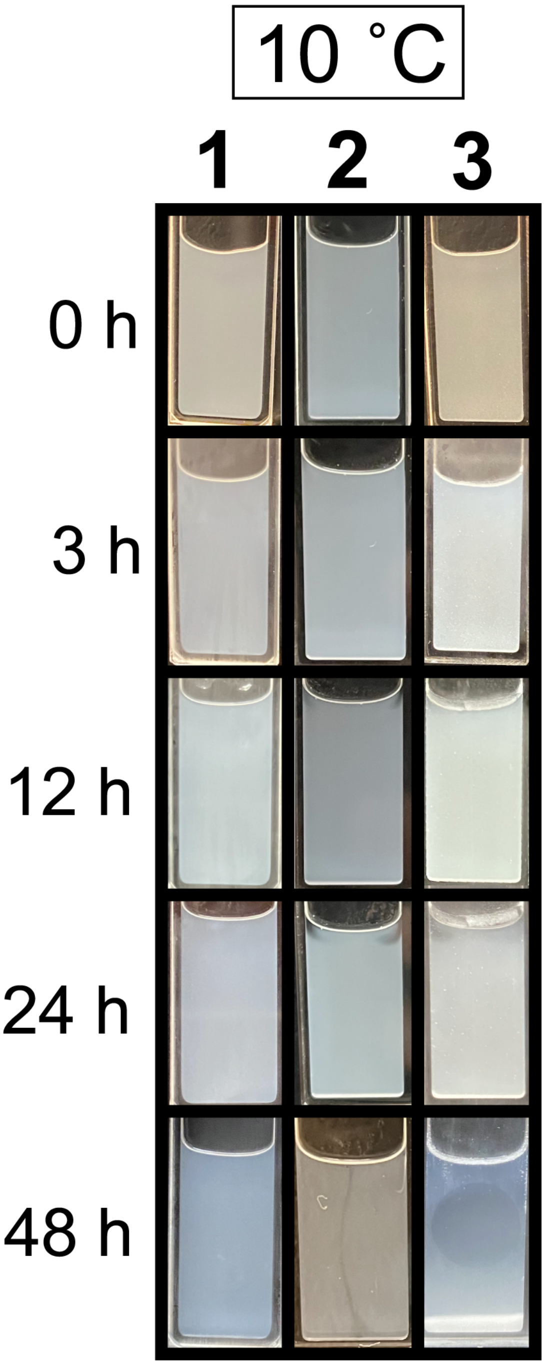

Figure S25. Pictures of dispersions of compound **1, 2,** and **3** in water containing Pluronic F127 (15 μM) at 10 °C for the visual assessment of sample stability. Samples were prepared at room temperature and immediately incubated at 10 °C for 48 h. Pictures of samples were taken at 0, 3, 12, 24, 48 h.

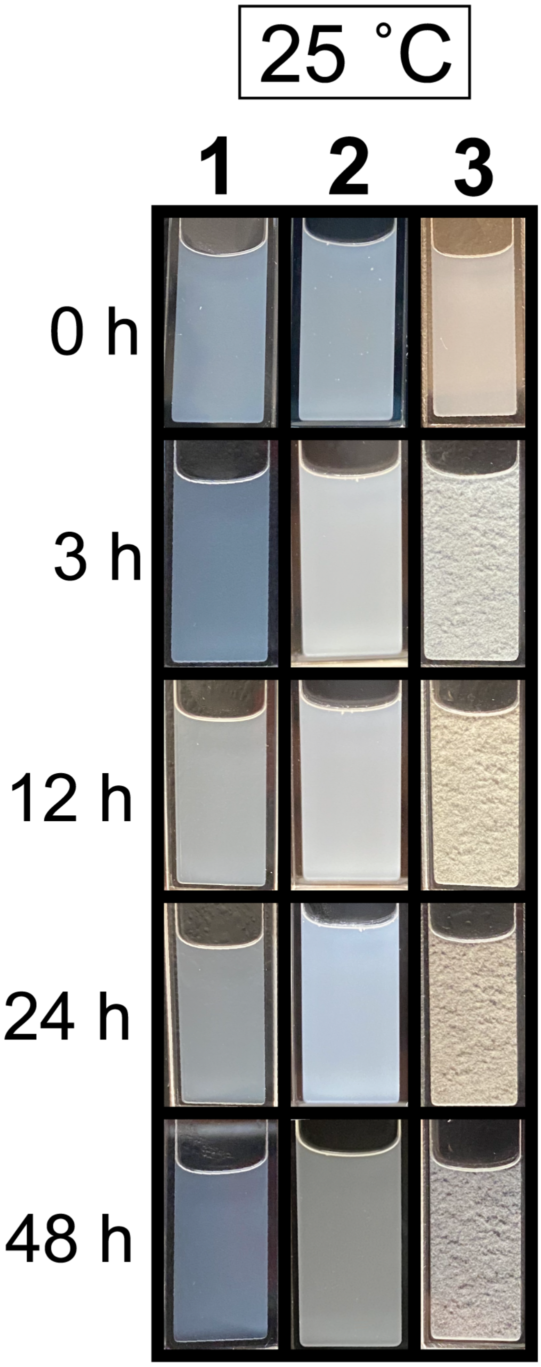

Figure S26. Pictures of dispersions of compound **1, 2,** and **3** in water containing Pluronic F127 (15 μM) at 25 °C for the visual assessment of sample stability. Samples were prepared at room temperature and immediately incubated at 10 °C for 48 h. Pictures of samples were taken at 0, 3, 12, 24, 48 h.

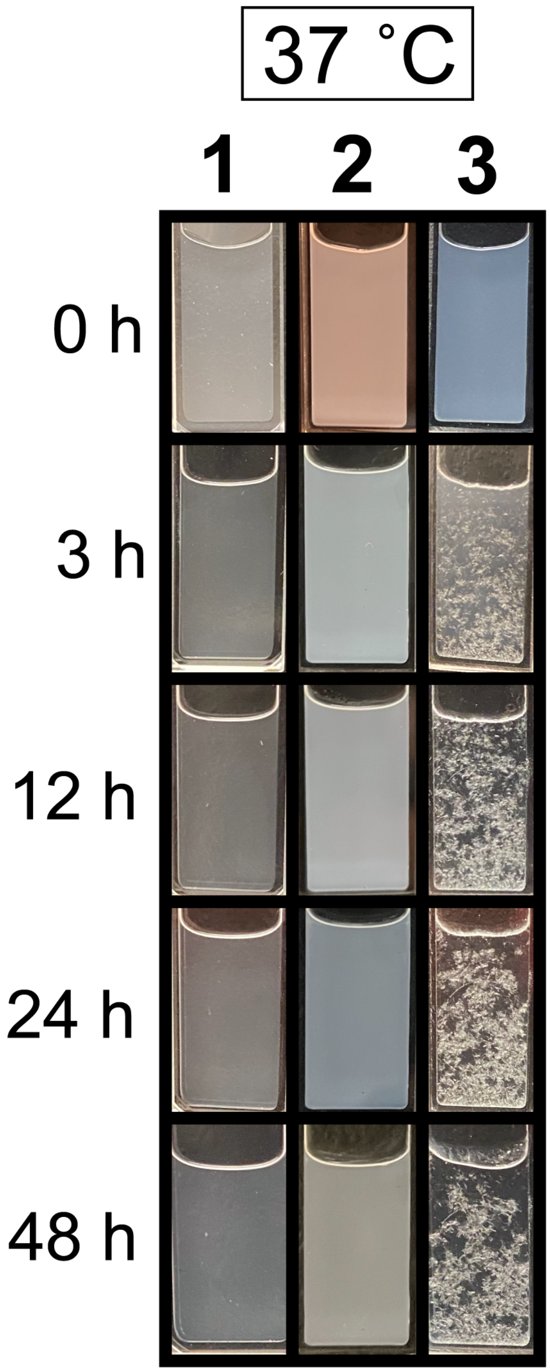

Figure S27. Pictures of dispersions of compound **1, 2,** and **3** in water containing Pluronic F127 (15 μM) at 37 °C for the visual assessment of sample stability. Samples were prepared at room temperature and immediately incubated at 10 °C for 48 h. Pictures of samples were taken at 0, 3, 12, 24, 48 h.

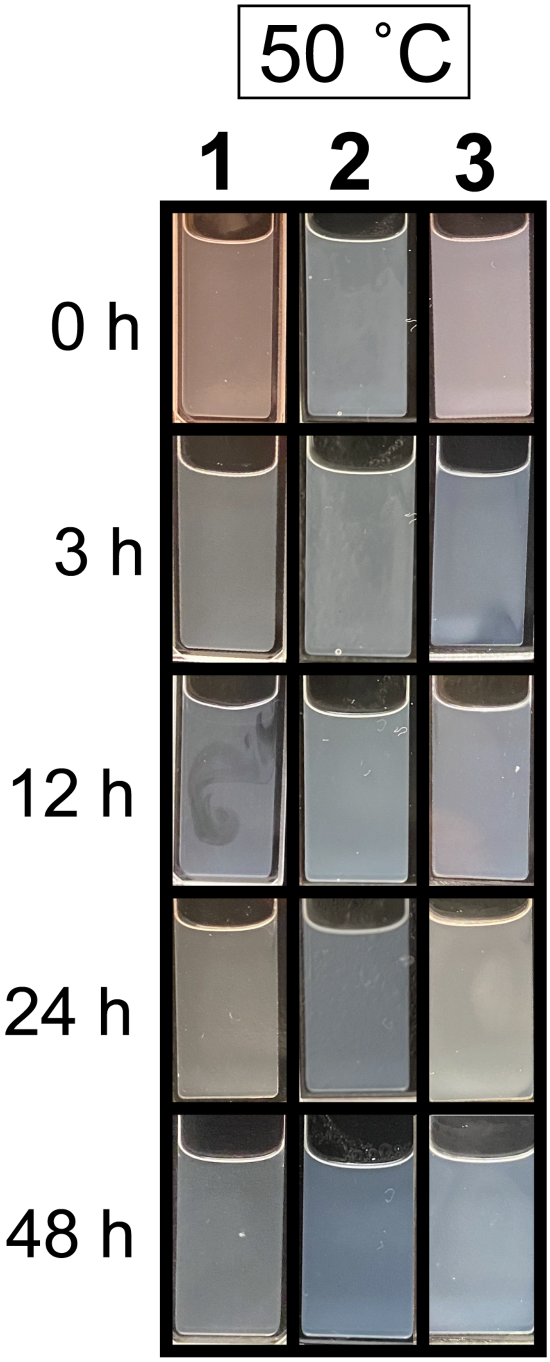

Figure S28. Pictures of dispersions of compound **1, 2,** and **3** in water containing Pluronic F127 (15 μM) at 50 °C for the visual assessment of sample stability. Samples were prepared at room temperature and immediately incubated at 10 °C for 48 h. Pictures of samples were taken at 0, 3, 12, 24, 48 h.

2.7 Fourier-transform IR (FTIR) spectroscopy

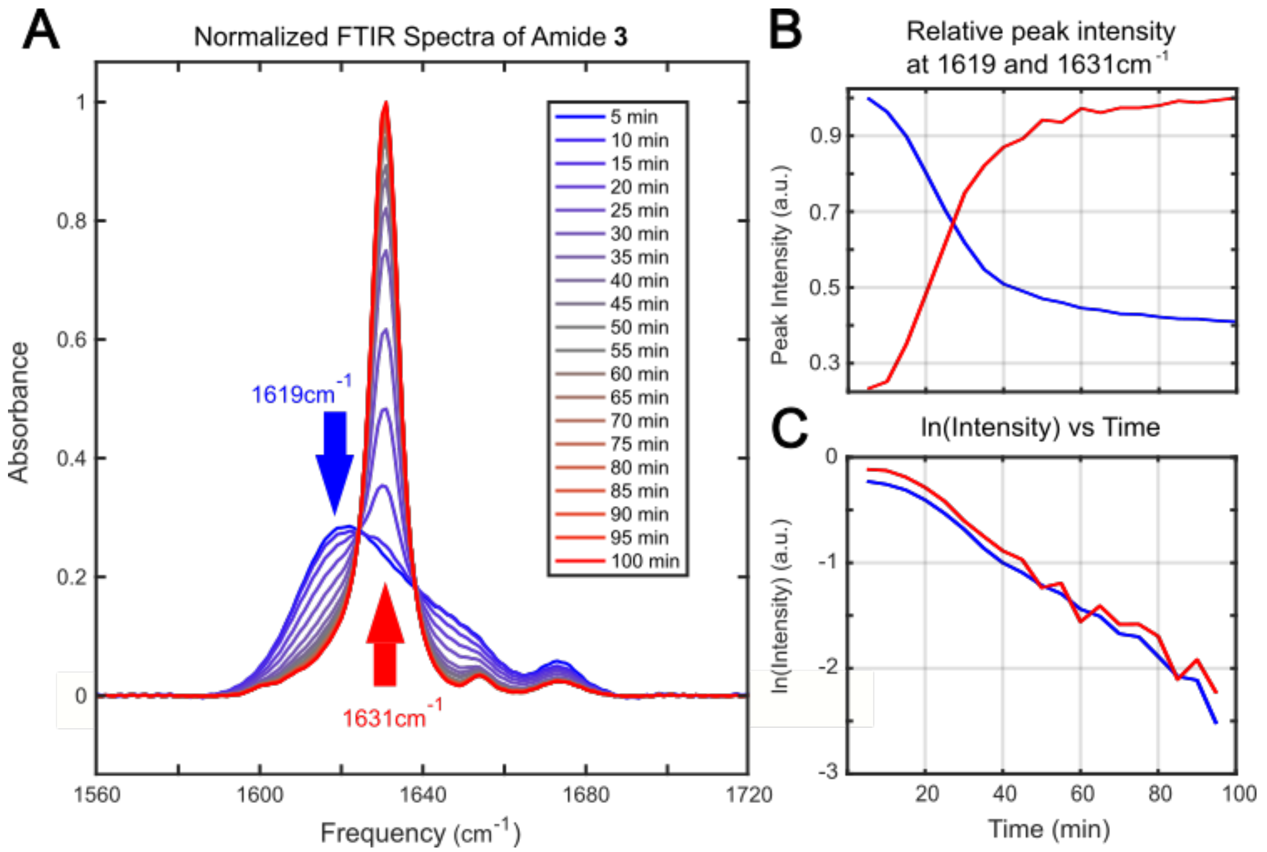

Figure S29. Time-dependent FTIR of a dispersion of compound **3**. **A)** Normalized FTIR spectra in the carbonyl region taken from 5 minutes to 100 minutes. Carbonyl 1HB stretch at 1619 cm^{-1} is noted by a blue arrow, and 0HB at 1631 cm^{-1} is noted by a red arrow. **B)** The blue and red lines represent relative absorbance values of 1619 and 1631 cm^{-1}, respectively. **C)** The natural logarithm of absorbance at 1619 cm^{-1} (blue) and the natural logarithm of (1 – peak intensity) at 1631 cm^{-1} were plotted for comparison purposes. The time constant of compound **3** precipitation as a first-order reaction is 40 minutes.

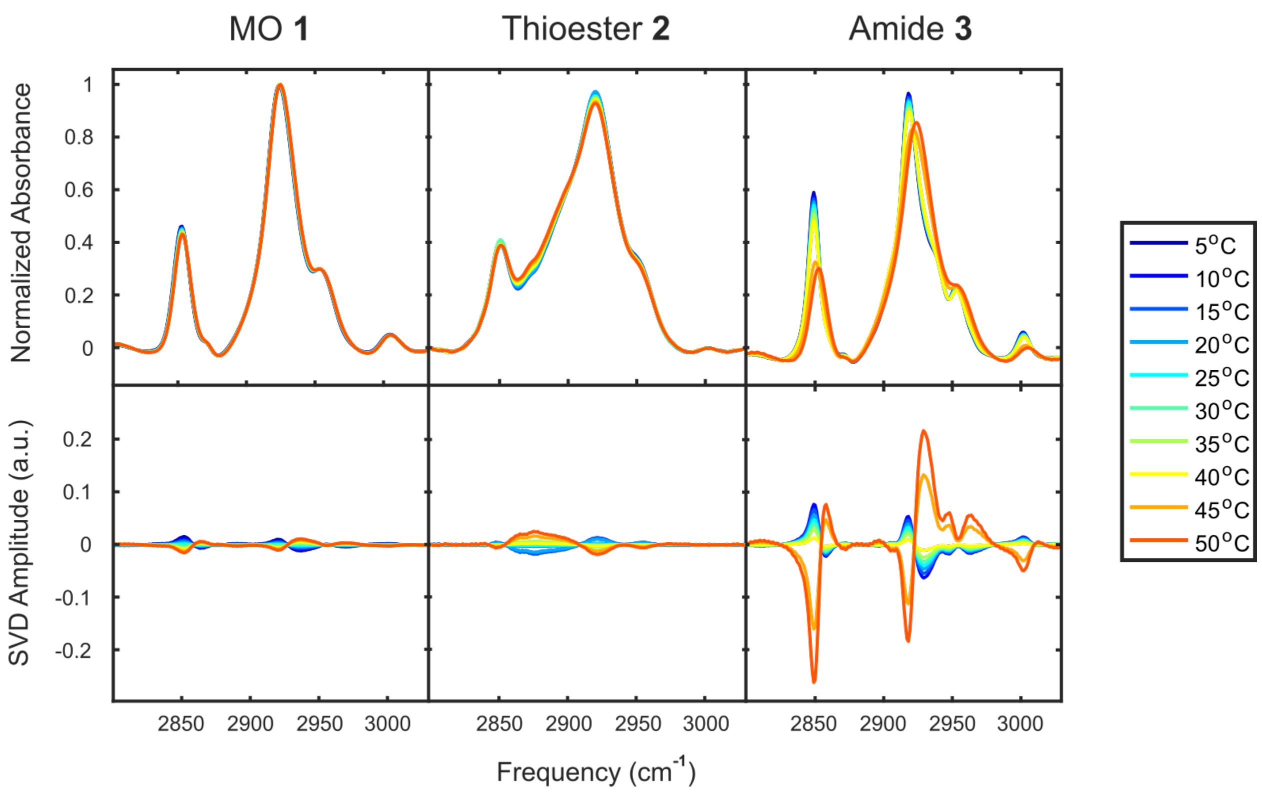

Figure S30. Temperature-dependent FTIR spectra of the CH_2 symmetric stretch measured from 5 °C to 50 °C. The upper panels show the normalized absorbance, and the lower panels show the second component of the singular-value-decomposition (SVD) of the spectra. For compounds **3**, the stretch band centered around 2925 cm^{-1} shows a clear blue shift at higher temperatures, starting at 45 °C). The shift of MO **1** and thioester **2** is insignificant compared to the amide **3** analog.

2.8 Electronic structure models

The initial MO configuration is obtained from the CHARMM-GUI website: CHARMM Small Molecule Library (CSML) monoolein-1.[2] To understand the impact of the chemical differences, the original structure was modified accordingly for the amide and thioester analogs. The geometry optimization and vibrational frequencies of the carbonyl groups were computed at the BP86/SDD level of theory using the Gaussian16 package of programs.[3–5] Vibrational frequencies and transition dipole moments extracted from harmonic frequency calculations were convolved with a Gaussian function to generate the spectra shown in Figure 4A. A frequency scaling factor of 1.021 was applied based on the database of frequency scale factors.[6]

3. References

(1) Osornio, Y. M.; Uebelhart, P.; Bosshard, S.; Konrad, F.; Siegel, J. S.; Landau, E. M. Design and Synthesis of Lipids for the Fabrication of Functional Lipidic Cubic-Phase Biomaterials. *J. Org. Chem.* **2012**, *77* (23), 10583–10595.
(2) Jo, S.; Kim, T.; Iyer, V. G.; Im, W. CHARMM-GUI: A Web-Based Graphical User Interface for CHARMM. *J. Comput. Chem.* **2008**, *29* (11), 1859–1865.
(3) Dunning, T. H.; Hay, P. J. Gaussian Basis Sets for Molecular Calculations. In *Modern Theoretical Chemistry*; Schaefer, H. F., Ed.; Springer: Boston, USA, 1977.
(4) Becke, A. D. Density-Functional Exchange-Energy Approximation with Correct Asymptotic Behavior. *Phys. Rev. A* **1988**, *38* (6), 3098–3100.
(5) Perdew, J. P. Density-Functional Approximation for the Correlation Energy of the Inhomogeneous Electron Gas. *Phys. Rev. B* **1986**, *33* (12), 8822–8824.
(6) Kanchanakungwankul, S.; Bao, J. L.; Zheng, J.; Alecu, I. M.; Lynch, B. J.; Zhao, Y.; Truhlar, D. G. Database of Frequency Scale Factors for Electronic Model Chemistries −Version 5.
